# *Drosophila* dendrites are resilient to adult-onset deficits in mitochondrial dynamics

**DOI:** 10.1083/jcb.202511010

**Published:** 2026-10-19

**Authors:** Hailey Q. Wang, Demetria G. Fortson, Eavan D. Burton, Hunter S. Whitbeck, Briana M. Robles, Kwass Wass, Letitia Bortey, Avi J. Adler, Jordan I. Kalai, Giovanna Napoleone, Erin L. Barnhart

**Affiliations:** 1 Department of Biological Sciences, Columbia University, New York, NY, USA

## Abstract

Mitochondrial transport, fusion, and fission are necessary for neuronal development, but the role of mitochondrial dynamics in neuronal maintenance remains unclear. In this work, we employed functional *in vivo* imaging of neurons in the *Drosophila* visual system, “horizontal system” (HS) cells, to determine how adult-onset deficits in mitochondrial dynamics affect mitochondrial localization, local regulation of ATP, and dendrite maintenance. In mature HS neurons, inhibition of mitochondrial transport or fusion depleted mitochondria from the dendrite over time but, surprisingly, had no effect on dendrite morphology. Moreover, adult-restricted mitochondrial mislocalization affected neither visual stimulus-driven dendritic calcium responses nor local, dynamic regulation of ATP levels. In contrast, when induced during development, the same perturbations caused mitochondrial mislocalization, loss of dendrite complexity, abrogation of stimulus-locked calcium responses and ATP fluctuations, and age-dependent dendrite degeneration. Thus, although mitochondrial dynamics are necessary during neuronal development, mature dendrites are capable of maintaining form and function *in vivo* upon mitochondrial depletion.

## Teaser

Wang et al. measure and manipulate mitochondrial dynamics in neurons in the *Drosophila* visual system. In mature neurons, inhibition of mitochondrial transport or fusion depletes mitochondria from the dendrite but has no effect on dendrite morphology, visual responses, or local, dynamic regulation of ATP levels.

## Introduction

Power-hungry neurons are widely believed to rely on proper localization of healthy mitochondria to coordinate ATP production with fluctuating energetic demands (Devine and Kittler, 2018; Misgeld and Schwarz, 2017; Sheng, 2017). Maintenance of properly localized, healthy mitochondria, or mitostasis, is particularly challenging in neurons (Misgeld and Schwarz, 2017). Mitochondria and their constituent elements (e.g., mitochondrial proteins) have substantially shorter lifespans than neurons (Vincow et al., 2013), and the vast majority of mitochondrial proteins are encoded by the nuclear genome. Therefore, neurons are thought to rely on mitochondrial motility to distribute mitochondria supplied with newly synthesized proteins to axonal and dendritic arbors far removed from the cell soma (Misgeld and Schwarz, 2017). Mitochondrial fusion and fission, in turn, are thought to contribute to mitostasis by facilitating the equitable distribution of functional mitochondrial components throughout the mitochondrial network (via fusion-dependent exchange of mitochondrial contents), as well as selective degradation of damaged mitochondria (via fission-dependent segregation) (Chan, 2020). According to this model, adult-onset deficits in mitochondrial dynamics ought to disrupt mitostasis and thus local production of ATP in neurites, ultimately resulting in neurodegeneration. However, although there is abundant evidence that mitochondrial motility, fusion, and fission are necessary for neuronal development (Chen et al., 2007; Courchet et al., 2013; Dickey and Strack, 2011; Dubal et al., 2022; Fukumitsu et al., 2015; Ishihara et al., 2009; Iwata et al., 2020; Kochan et al., 2024; Li et al., 2004; López-Doménech et al., 2016; Morris and Hollenbeck, 1993; Tröger et al., 2025), the extent to which mitochondrial dynamics contribute to maintenance of mature neurons *in vivo* remains unknown.

The role of mitochondrial dynamics in neuronal maintenance is poorly defined for several reasons. First, it is unclear how much mitochondrial transport, fusion, and fission occur in fully mature neurons *in vivo*. Mitochondrial transport has been measured in neurons *in vivo* (Donovan et al., 2024; Mandal et al., 2021; Plucińska et al., 2012; Silva et al., 2021; Vagnoni and Bullock, 2018), but in some cell types, transport declines dramatically as neurons mature (Faits et al., 2016; Lewis et al., 2016; Smit-Rigter et al., 2016), suggesting that mitochondrial motility may be more important for neuronal development than for neuronal maintenance. Moreover, although mitochondrial fusion, fission, and content exchange have been observed in neurons *ex vivo* (Fenton et al., 2024; Kochan et al., 2024; Lewis et al., 2018), content exchange has yet to be measured in neurons *in vivo*. Second, deficits in mitochondrial dynamics reduce neuronal morphological complexity (Berthet et al., 2014; Chen et al., 2007; Courchet et al., 2013; Dickey and Strack, 2011; Fukumitsu et al., 2015; Ishihara et al., 2009; López-Doménech et al., 2016; Richhariya et al., 2025) and disrupt synaptic plasticity (Divakaruni et al., 2018; Li et al., 2004) and synaptic transmission (Lewis et al., 2018; Verstreken et al., 2005; Vevea and Chapman, 2023). However, with limited exceptions (Berthet et al., 2014; Chen et al., 2007; López-Doménech et al., 2016), these mitochondrial deficits have been examined either in cell culture or in the context of genetic manipulations that affect neuronal development and/or large populations of neurons *in vivo*, making it difficult to determine whether neuronal dysfunction is due to cell-intrinsic defects in mitostasis that disrupt neuronal maintenance versus developmental effects that disrupt neuronal growth. Indeed, when induced in midlife, pan-neuronal inhibition of mitochondrial fusion or fission, rather than causing neuronal degeneration, results in lifespan extension in *Drosophila* (Rana et al., 2017). Lastly, experiments in cultured neurons have shown that mitochondrial oxidative phosphorylation (OXPHOS) is necessary for activity-dependent upregulation of ATP production (Rangaraju et al., 2014), but there is no direct evidence linking cell type–specific deficits in mitochondrial transport, fusion, or fission to disrupted energy homeostasis in neurons *in vivo*. Altogether, it is necessary to define the cell-intrinsic effects of disrupted mitochondrial dynamics on neuronal maintenance.

**Figure 1.**
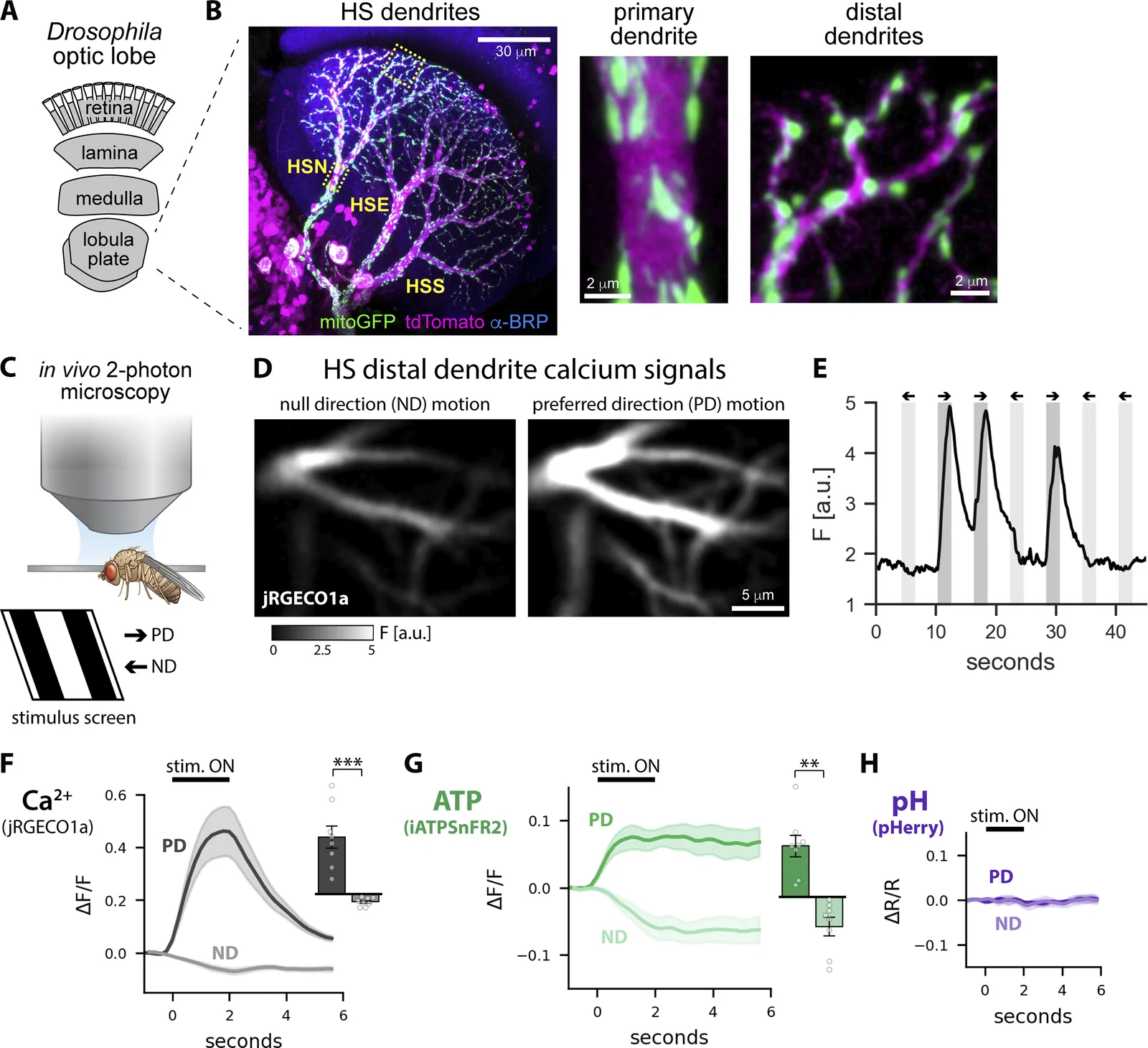
**Visual input drives ATP responses in HS dendrites. (A)** Organization of the *Drosophila* visual system. **(B)** Representative image of the dendrites of 3 HS neurons in the optic lobe (HSN, HSE, and HSS). The neurons were labeled with a cytosolic volume marker (tdTomato, magenta) and a mitoGFP (green); the neuropil was labeled by immunostaining for the synaptic marker BRP (α-BRP, blue). Dashed yellow boxes indicate the primary and distal dendrites shown on the right. All images are maximum projections of z-stacks acquired using confocal Airyscan imaging. **(C)** Experimental setup. *In vivo* two-photon imaging of HS dendrites in a head-fixed fly with simultaneous visual stimulus presentation. The visual stimulus—full-contrast square-wave gratings that spanned the entire stimulus screen—moved in either the PD for HS neurons (front-to-back, with respect to the fly’s eye) or the opposite direction, ND. **(D)** Two-photon images (average projections of a time series) of cytosolic calcium (jRGECO1a) in HS distal dendrites during presentation of gratings moving in the ND (left image) or PD (right image). **(E)** Example jRGECO signals, plotted over time, in response to moving gratings. Shaded regions on the graph indicate when the grating was moving; light gray indicates ND motion, and dark gray indicates PD motion. Two-second bouts of motion were interspersed with four-second presentations of static gratings. **(F and G)** Average calcium (jRGECO1a, F) and ATP (iATPSnFR2, G) responses to ND and PD motion, plotted over time. Insets show average response amplitudes. Shading on line plots and error bars on bar plots indicate the standard error of the mean; dots overlaid on the bar plots indicate average response amplitudes for individual flies (*N* = 9 flies). Asterisks indicate significant differences (**P < 0.01, ***P < 0.001, paired *t* test). **(H)** Average change in pH (pHerry) in response to the ND and PD motion, plotted over time (*N* = 3 flies).

In this work, we employed *Drosophila* genetic tools and functional *in vivo* imaging to measure and manipulate mitochondrial dynamics in a cell type–specific and temporally restricted fashion. We focused on neurons in the fly visual system called “horizontal system” (HS) cells. There are 3 HS neurons per optic lobe, each of which has an elaborately branched dendrite that innervates the third neuropil of the visual system, the lobula plate (Fig. 1, A and B). HS dendrites are densely packed with mitochondria (Fig. 1 B) (Donovan et al., 2024), and their morphologies and functional properties are well defined (Barnhart et al., 2018; Busch et al., 2018; Cuntz et al., 2008; Erginkaya et al., 2025; Kim et al., 2017; Krapp et al., 1998; Schnell et al., 2010; Scott et al., 2002). HS neurons are global motion detectors that selectively respond to specific patterns of global optic flow evoked by self-motion of the fly. Specifically, HS neurons depolarize in response to a preferred direction (PD) of visual global motion (front-to-back with respect to the fly’s eye) and hyperpolarize in response to global motion in the opposite, null direction (ND, back-to-front with respect to the fly’s eye) (Schnell et al., 2010). HS direction selectivity is mediated by excitatory and inhibitory input onto HS distal dendrites: local motion detectors activated by front-to-back motion provide direct excitatory input, while inhibitory interneurons relay input from neurons activated by local motion in the opposite direction (Maisak et al., 2013; Mauss et al., 2015; Nern et al., 2025). In addition, we demonstrate here that PD global motion drives sustained increases in ATP levels in HS dendrites, making them ideal for interrogating the link between mitochondrial dynamics, local ATP production, and the maintenance of neuronal form and function. By measuring mitochondrial motility and content exchange in HS dendrites in the adult, we show that mitochondria in fully developed HS neurons are in fact dynamic. We further demonstrate that inhibiting mitochondrial transport or fusion in HS neurons during development causes mitochondrial mislocalization and/or fragmentation, reduced dendrite complexity, and age-dependent dendrite degeneration. In contrast, when restricted to the adult, the same perturbations cause depletion of mitochondria from the dendrite over time, but have no measurable effects on dendrite morphology, stimulus-driven ATP responses, or the direction selectivity of dendritic calcium or voltage responses. Thus, HS dendrites are surprisingly resilient to adult-onset deficits in mitochondrial dynamics and localization.

## Results

### Visual input drives ATP responses in HS dendrites

**Figure S1.**
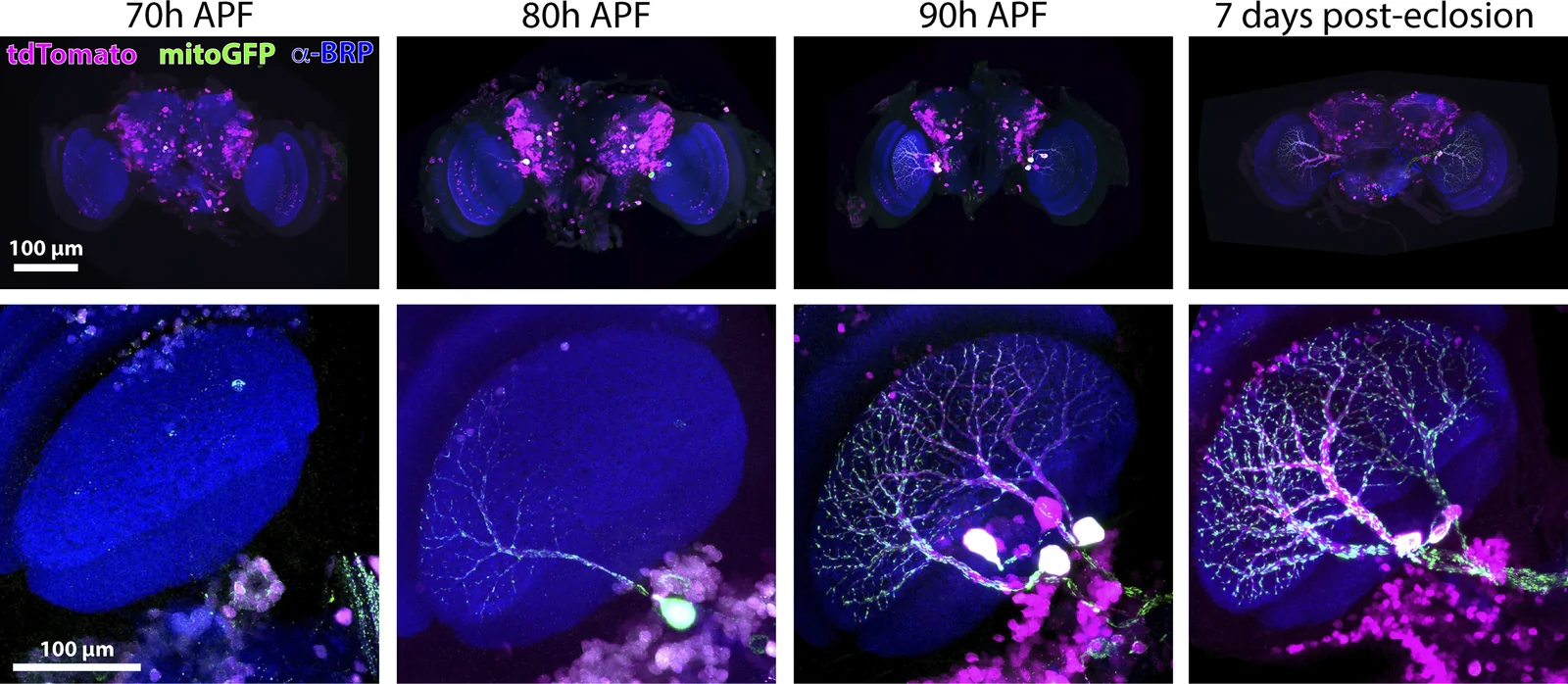
**R27B03-GAL4 driver line labels HS neurons in late pupation and adulthood.** Representative images of R27B03-GAL4 expression in whole *Drosophila* brains (top row) and the lobula plate of the optic lobe (bottom row) at 70, 80, or 90 h APF and 7 days after eclosion. Neurons were labeled by GAL4-driven expression of tdTomato (magenta) and mitoGFP (green), and the neuropil was labeled by immunostaining for the synaptic marker BRP (blue). All images are maximum projections of z-stacks acquired using confocal Airyscan imaging. APF, after pupal formation.

To investigate how deficits in mitostasis affect local, dynamic regulation of ATP levels, we first set out to measure visual stimulus-driven ATP fluctuations in HS dendrites. In cell culture, neurons upregulate ATP production in an activity-dependent fashion (Rangaraju et al., 2014). In *Drosophila*, stochastic fluctuations in neuronal calcium signals correlate with fluctuations in ATP across brain regions, suggesting that neuronal activity also upregulates ATP production *in vivo* (Mann et al., 2021). To determine whether neuronal activation affects ATP levels in HS dendrites, we expressed genetically encoded fluorescent reporters for ATP (iATPSnFR2 [Marvin et al., 2024]) and calcium (jRGECO1a [Dana et al., 2016]) in HS neurons using the GAL4/UAS system. We used a GAL4 driver line that drives expression in HS neurons in late pupal stages, as well as throughout adulthood, with expression limited to HS neurons in the optic lobe (Fig. S1), and we imaged HS dendrites in head-fixed flies via two-photon microscopy while simultaneously projecting visual stimuli on a screen positioned in front of the fly (Fig. 1 C). For these functional imaging experiments, we imaged distal dendritic branches, which receive the majority of synaptic inputs to the dendrite (Nern et al., 2025). We presented full-contrast square-wave gratings that spanned the entire stimulus screen (∼60 × 60°) and moved in either the PD or ND. Two-second bouts of global motion were interspersed with four-second periods without motion (i.e., presentation of a static grating), and bouts of PD versus ND motion were presented in a randomized order. As expected, based on previously published work (Barnhart et al., 2018), we measured direction-selective calcium responses to global motion: PD motion drove large increases in jRGECO1a fluorescence, whereas ND motion triggered a slight reduction in fluorescence (Fig. 1, D–F).

Strikingly, we also measured stimulus-locked fluctuations in ATP levels (Fig. 1 G). PD motion drove a sustained increase in iATPSnFR2 fluorescence, suggesting excitatory input onto HS dendrites triggers upregulation of ATP synthesis, with ATP production outpacing any stimulus-driven increase in ATP consumption. In contrast, ND motion caused a sustained reduction in fluorescence, suggesting inhibitory input increases ATP consumption without triggering a commensurate increase in ATP production. iATPSnFR2 is pH-sensitive, so to determine whether visual input causes fluctuations in pH in HS distal dendrites, we expressed a genetically encoded pH sensor, pHerry (Rossano et al., 2017), in HS neurons. pHerry consists of a pH-sensitive green fluorescent protein (SE-pHluorin) fused to a pH-insensitive red fluorescent protein (mCherry). Neither PD nor ND motion drove a change in the pHerry green-to-red fluorescence ratio (Fig. 1 H), suggesting that our measurements of stimulus-driven fluctuations in iATPSnFR2 fluorescence reflect changes in ATP levels rather than changes in pH. Our ability to measure stimulus-locked fluctuations in ATP levels makes HS dendrites an ideal model for investigating how deficits in mitostasis affect dynamic regulation of ATP in a physiological context.

### Mitochondrial transport and content exchange in HS dendrites

**Figure 2.**
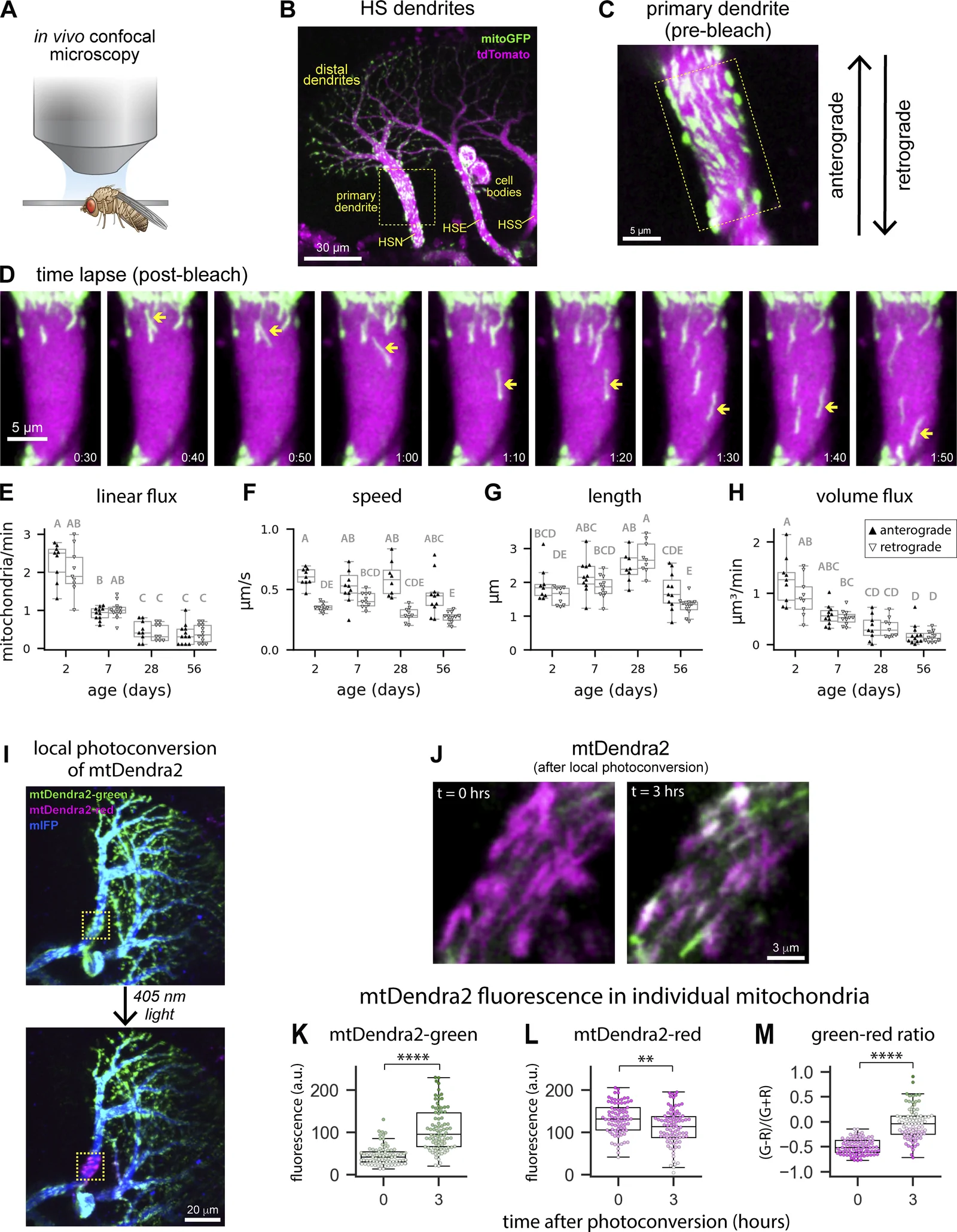
**Mitochondrial dynamics in HS dendrites. (A)** Experimental setup. *In vivo* confocal imaging of head-fixed flies. **(B and C)** Confocal images of mitochondria (mitoGFP, green) in HS dendrites (tdTomato, magenta) before photobleaching of mitochondria in the primary dendrite. The dashed yellow box in B indicates the primary dendrite shown in D. **(D)** Image time series showing mitochondrial transport in the primary dendrite shown in C. To facilitate resolution of motile mitochondria, stationary mitochondria in the field of view were photobleached prior to image acquisition. The yellow arrow indicates a mitochondrion moving in the retrograde direction. Time (min:sec) is indicated in the bottom right corner of each image. **(E–H)** Box plots show the number of motile mitochondria moving through the primary dendrite per minute (linear flux, E), average pause-free speeds (F), lengths of motile mitochondria (G), and the total volume of mitochondria transported through the primary dendrite per minute (volume exchange, H), plotted as a function of fly age. Dots overlaid on the box plots indicate average values for individual flies (*N* = 9–11 flies per age), and significant differences among groups (Kruskal–Wallis test with post hoc Dunn’s test, P < 0.05) are indicated by the letters above each box; groups labeled with different letters are significantly different, and groups labeled with the same letter are not significantly different (see Statistical analysis in Materials and methods). **(I)***In vivo* confocal images of Dendra2-tagged mitochondria in HS dendrites before (top image) and after (bottom image) local photoconversion of mitochondria in the primary dendrite. **(J)** Dendra2-tagged mitochondria in a primary HS dendrite (labeled with cytosolic mIFP, blue) immediately after photoconversion (left image) and 3 h later (right image). **(K–M)** Quantification of mtDendra2-green fluorescence (K), mtDendra2-red fluorescence (L), and a metric quantifying the green-to-red ratio (M) in individual mitochondria in primary HS dendrites after photoconversion. The green-to-red ratio is given by (G−R)/(G+R), where G is the green fluorescence intensity, and R is the red fluorescence intensity. Dots overlaid on the box plots indicate measurements from manually segmented mitochondria; dot color reflects the fluorescence intensity of each mitochondrion (K and L) or the ratio of green to red fluorescence within each mitochondrion (M). Asterisks indicate significant differences (Mann–Whitney U test; **P < 0.001, ****P < 10^−5^); *N* = 87 mitochondria (t = 0 h) or 92 mitochondria (t = 3 h) from six neurons from six flies. All box-and-whisker plots (in this figure and all subsequent figures) show the median, interquartile range (box), and 1.5 times the interquartile range (whiskers).

Having established that visual input drives fluctuations in ATP in HS dendrites *in vivo*, we next wished to quantify mitochondrial dynamics in the same system. In previous work, we measured substantial mitochondrial transport in HS dendrites in young flies (Donovan et al., 2024). However, in some cell types, mitochondrial transport declines to nearly undetectable levels as neurons mature (Faits et al., 2016; Lewis et al., 2016; Smit-Rigter et al., 2016), casting some doubt on the extent to which mitochondrial transport contributes to mitostasis in adult neurons. To determine whether mitochondrial transport in HS dendrites continues throughout adulthood, we expressed a mitochondria-targeted GFP (mitoGFP) and a cytosolic volume marker (tdTomato) in HS neurons, and we used *in vivo* confocal microscopy to image motile mitochondria in head-fixed flies ranging from 2 days to 2 mo old (Fig. 2, A–H). To resolve individual motile mitochondria, we photobleached stationary mitochondria in the primary dendrite before collecting time-lapse images (Fig. 2, B–D). We imaged primary, rather than distal, HS dendrites because all motile mitochondria that enter or exit the dendritic arbor must move through the primary dendrite, and in previous work (Donovan et al., 2024), we found that motile mitochondria move persistently across branch points within the arbor, either from the parent branch to a daughter branch (anterograde) or from a daughter to the parent (retrograde) rather than between daughters. We therefore assume that transport through the primary dendrite, and any age-dependent changes thereof, reflects transport patterns throughout the dendrite.

Consistent with our previous work (Donovan et al., 2024), we observed mitochondria moving through the primary dendrite in both the anterograde direction (away from the cell body, into the dendritic arbor) and the retrograde direction (out of the arbor, back toward the cell body). At all ages, we measured balanced bidirectional transport (Fig. 2, E, G, and H). Although anterograde mitochondria moved at significantly higher speeds than retrograde mitochondria (Fig. 2 F), there was no difference in linear flux rates (the number of motile mitochondria moving through the primary dendrite per minute) or mitochondrial lengths for anterograde versus retrograde mitochondria (Fig. 2, E and G). Thus, at any given age, equivalent volumes of mitochondria move in the anterograde and retrograde directions per unit time (Fig. 2 H). Comparing across ages, however, we measured a significant reduction in linear flux rates over time, with linear flux rates rapidly declining in early adulthood before plateauing in older flies (Fig. 2 E). In addition, we measured age-dependent changes in the size of the motile mitochondria, with mitochondrial lengths increasing with age up until 28 days before decreasing again in 56-day-old flies (Fig. 2 G). This slight increase in mitochondrial size was not sufficient to compensate for the reduction in linear flux rates, however; overall, the total volume of mitochondria that exchanged through the primary dendrite declined with age (Fig. 2 H), with an approximately fivefold reduction in 56-day-old flies (volume exchange rate = ∼0.2 μm^3^/min) compared with 2-day-old flies (∼1 μm^3^/min).

In the simplest mitostasis model, mitochondria carrying fresh proteins move in the anterograde direction, away from the soma, and replace old mitochondria in neurites, which, in turn, are either degraded in place or trafficked back to the soma for degradation. Could the relatively low rates of mitochondrial transport in old flies impair the neuron’s ability to replenish mitochondria in distal dendrites? Any mitochondrion that moves into or out of the dendrite must pass through the primary dendrite, so we used our measurements of mitochondrial volume exchange (Fig. 2 H), along with previously reported estimates of the total mitochondrial volume in the dendritic arbor (∼400 μm^3^) (Donovan et al., 2024), to estimate mitochondrial replacement timescales—i.e., the amount of time necessary to replace the entire volume of mitochondria in the dendrite, based on transport alone—as a function of fly age (see Materials and methods). Even at reduced mitochondrial volume exchange rates in old flies (∼0.2 μm^3^/min or ∼12 μm^3^/h), we estimated a replacement timescale (∼1–2 days) that is substantially shorter than the median half-life for mitochondrial proteins in *Drosophila* (∼2 wk) (Vincow et al., 2013). Thus, reduced mitochondrial transport rates in old flies are unlikely to limit the rate of mitochondrial replenishment in HS dendrites.

**Figure S2.**
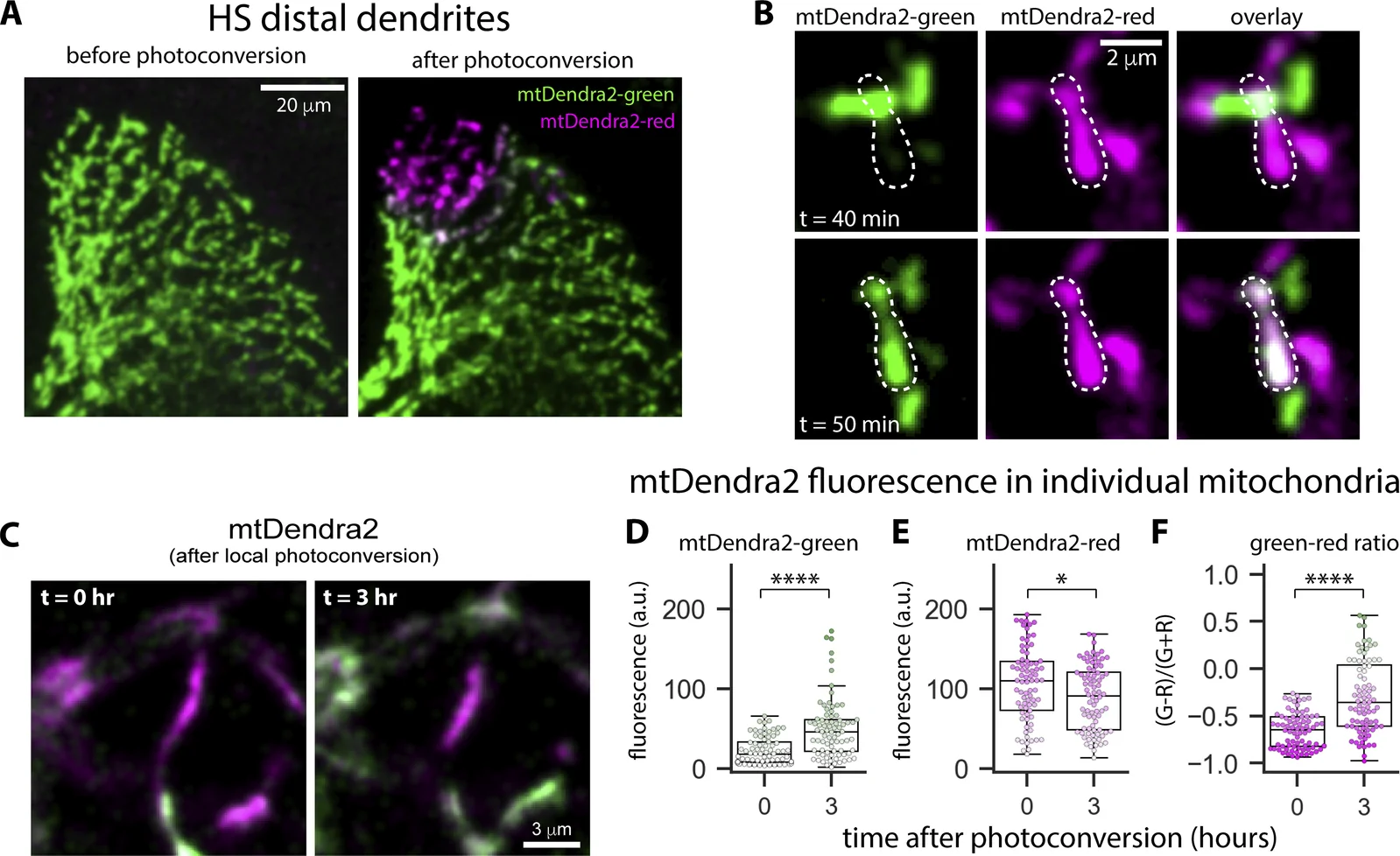
**Mitochondrial fusion and content exchange in HS distal dendrites. (A)** mtDendra2-labeled mitochondria in HS distal dendrites before (left image) and after (right image) local photoconversion. Images are max projections of confocal z-stacks. **(B)** Fusion between mitochondria labeled with mtDendra-green and mtDendra2-red. Images show the mitochondria before (t = 40 min, top row) and after fusion (t = 50 min, bottom row); the dashed line indicates a stationary mitochondrion, labeled with mtDendra-red, that receives mtDendra2-green. **(C)** Dendra2-tagged mitochondria in distal HS dendrites immediately after photoconversion (left image) and 3 h later (right image). **(D–F)** Quantification of mtDendra2-green fluorescence (D), mtDendra2-red fluorescence (E), and a metric quantifying the green-to-red ratio (F) in individual mitochondria in primary HS dendrites after photoconversion. The green-to-red ratio is given by (G-R)/(G+R), where G is the green fluorescence intensity, and R is the red fluorescence intensity. Dots overlaid on the box plots indicate measurements from manually segmented mitochondria; dot color reflects the fluorescence intensity of each mitochondrion (D–E) or the ratio of green to red fluorescence within each mitochondrion (F). Asterisks indicate significant differences (Mann–Whitney U test; *P < 0.05, ****P < 10^−5^); *N* = 78 mitochondria (t = 0 h) or 80 mitochondria (t = 3 h) from eight neurons from eight flies.

Having demonstrated that mitochondrial motility occurs at appreciable rates throughout adulthood, we next asked whether mitochondria in HS dendrites are also social, i.e., capable of fusing and dividing, as previously observed in neurons *ex vivo* (Fenton et al., 2024; Kochan et al., 2024; Lewis et al., 2018). To attempt to image mitochondrial fusion and fission events in HS dendrites, we combined *in vivo* confocal microscopy of young (7-day-old), head-fixed flies with HS-specific expression of a mitochondria-targeted photoconvertible fluorescent protein, mtDendra2. We converted a small population of Dendra2-tagged mitochondria from green to red fluorescence by locally illuminating a region of interest (ROI) in the dendrite with UV light, and then, we imaged the photoconverted ROI over time (Fig. 2 I; and Fig. S2, A and B). We achieved efficient green-to-red photoconversion of the mtDendra2, and we were able to directly observe a small number of fusion events (Fig. S2, A and B). However, rapid photobleaching of mtDendra-green prevented us from imaging at a high frame rate for an extended period of time, hampering our ability to reliably image discrete fusion and fission events and quantify fusion/fission rates. Instead, we measured mitochondrial content exchange in primary HS dendrites by quantifying green and red fluorescence within manually segmented mitochondria in the photoconverted ROI immediately after photoconversion and 3 h later (Fig. 2 J). We reasoned that if content exchange occurs in HS dendrites *in vivo*, then red mitochondria in the ROI should accumulate green fluorescence as mitochondria labeled with mtDendra2-green move into the ROI and fuse with stationary mitochondria labeled with mtDendra2-red. In contrast, if no exchange occurs and replenishment of mitochondria occurs via mitochondrial replacement alone—i.e., transport of new mitochondria into the dendrite balanced by transport of old mitochondria out of the dendrite—then green mitochondria should appear in the ROI and red mitochondria should disappear. We found, first, that mitochondria were primarily red immediately after photoconversion (Fig. 2, I, J, and M). Three hours later, the amount of green-to-red fluorescence significantly increased in mitochondria due to an increase in mtDendra2-green fluorescence, as well as a slight decrease in mtDendra2-red fluorescence (Fig. 2, K–M). Although a small number of mitochondria in the ROI 3 h after photoconversion were almost entirely green, consistent with mitochondrial replacement, the majority were both green and red, consistent with mitochondrial content exchange (Fig. 2, J and M). We measured a similar pattern of green fluorescence recovery in HS distal dendrites, albeit at a slower rate than in primary dendrites (Fig. S2, C–F). These results demonstrate that recovery of mtDendra2-green in HS dendrites primarily occurs via fusion-mediated mitochondrial content exchange rather than replacement of whole mitochondria. Thus, mitochondria in HS dendrites are social and motile.

### Inhibition of mitochondrial transport and content exchange during development and adulthood disrupts dendrite form and function

**Figure 3.**
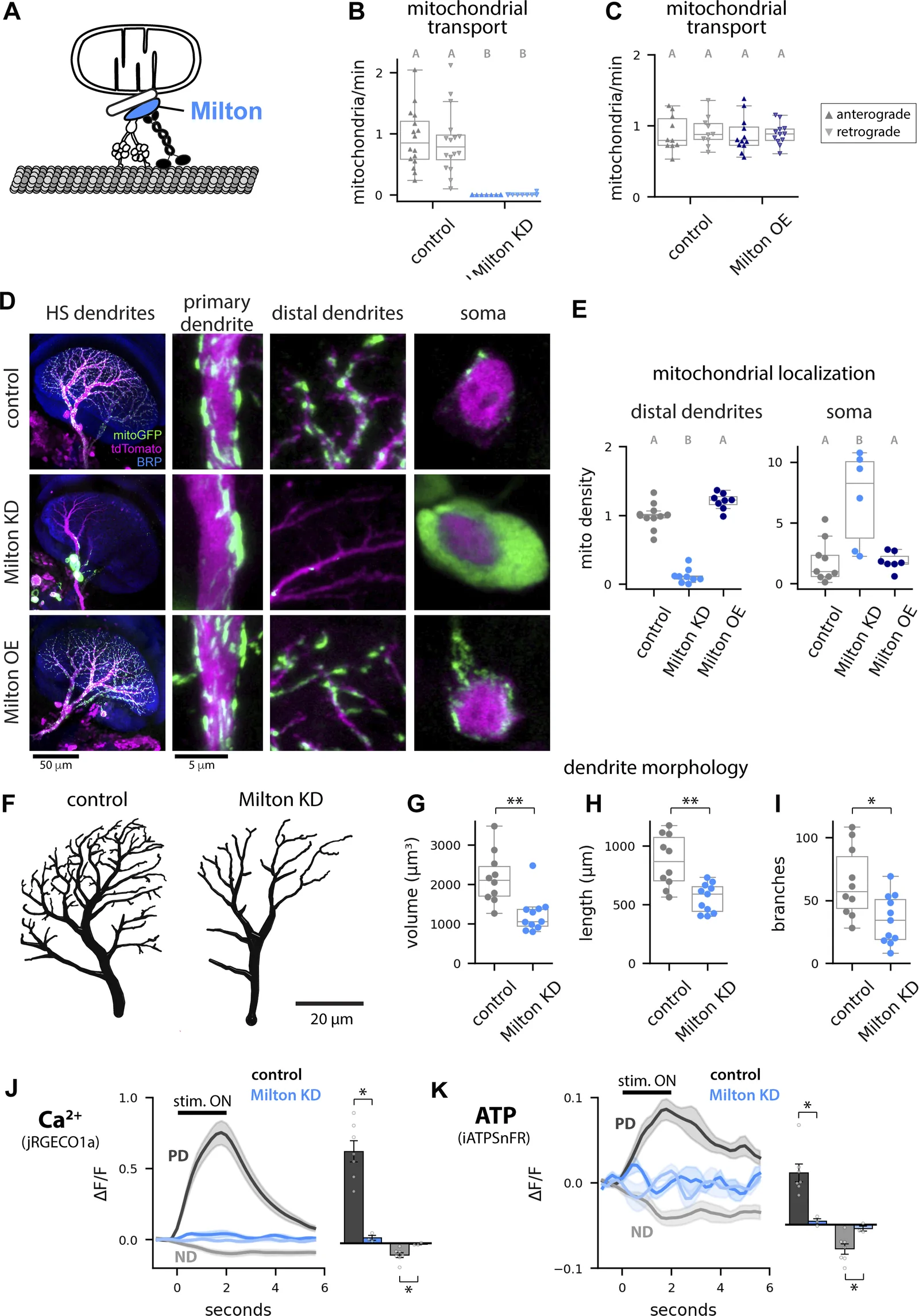
**Milton KD during development and adulthood causes mitochondrial mislocalization and disrupts dendrite form and function. (A)** An adaptor protein, Milton (bright blue), links mitochondria to microtubule motor proteins via direct binding to a mitochondrial outer membrane protein (Miro). **(B and C)** Mitochondrial linear flux rates in primary dendrites of control and Milton KD (B) or Milton overexpression (C) samples. Dots overlaid on the box plots indicate measurements for individual cells; *N* = 10–17 cells per genotype. Letters above each box indicate groups that are significantly different (Kruskal–Wallis test with post hoc Dunn’s test, P < 10^−4^). **(D)** Representative images of HS dendrites (labeled with cytosolic tdTomato, magenta) and the mitochondria within them (mitoGFP, green) in control (top row of images), Milton KD (middle row), and Milton overexpression samples (bottom row). Overview images on the left show all HS dendrites in the optic lobe; images of the primary and distal dendrites and somas are enlarged on the right. **(E)** Mitochondrial volume densities, normalized to the median of the control, in distal dendrites (left plot) and somas (right plot). Dots overlaid on box plots indicate average measurements for individual flies; *N* = 8–10 (distal dendrites) and 6–9 (somas) flies per genotype. Letters indicate significant differences (Kruskal–Wallis test with post hoc Dunn’s test, P < 0.01). **(F)** Representative dendrite reconstructions from control and Milton KD neurons. **(G–I)** Dendrite volume (G), dendrite length (H), and number of branches (I). Dots overlaid indicate measurements for individual dendrites; *N* = 10–11 dendrites per genotype. Asterisks indicate significant differences (Mann–Whitney U test; *P < 0.05, **P < 0.01). **(J and K)** Average calcium (jRGECO1a, J) and ATP (iATPSnFR, K) responses to wide-field square-wave gratings moving in the PD or ND in distal dendrites of control (gray, *N* = 7 flies) and Milton KD (blue, *N* = 3 flies) samples. Insets show average response amplitudes. Asterisks indicate significant differences (Mann–Whitney U test; *P < 0.05).

**Figure S3.**
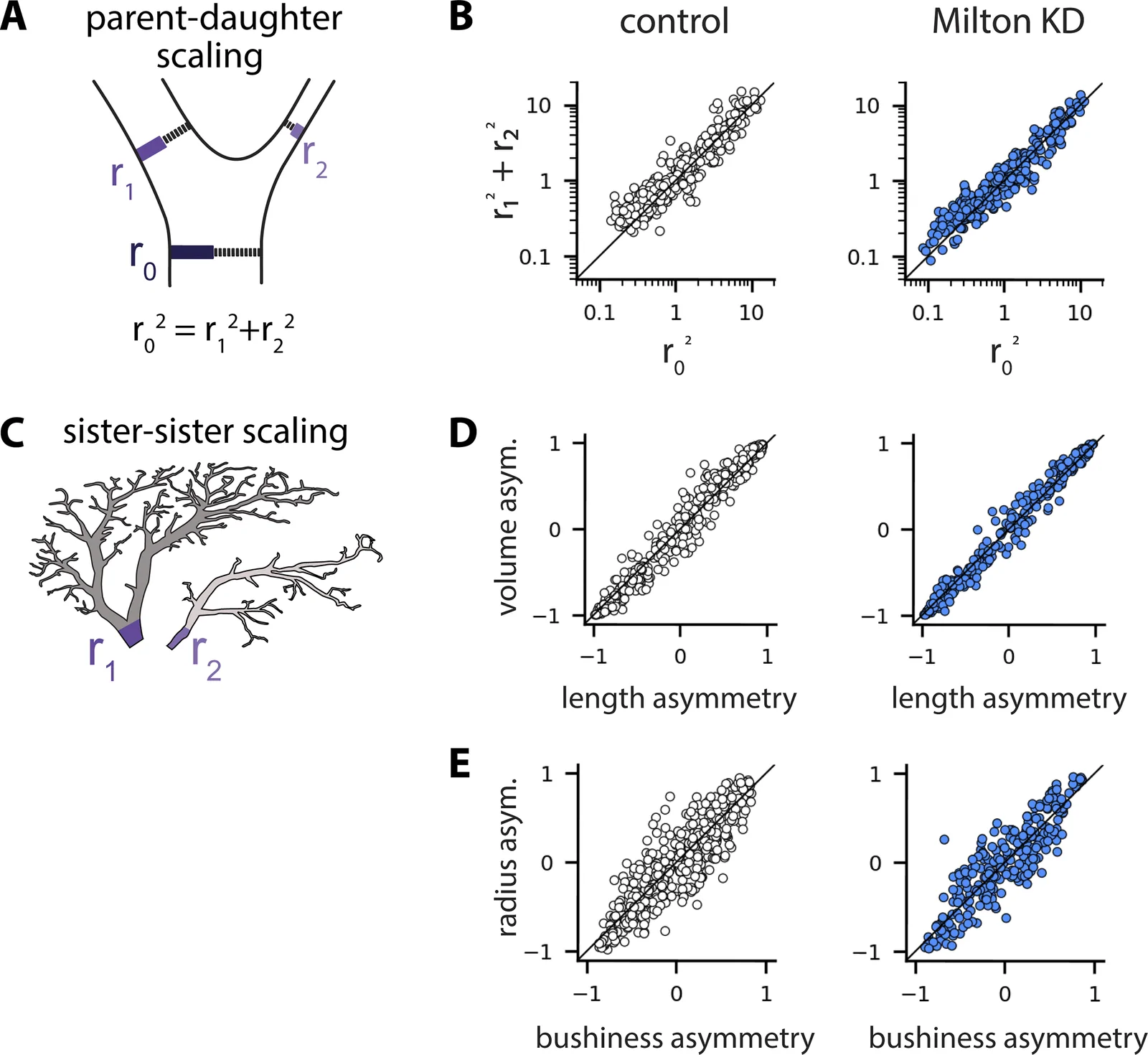
**Milton KD has no effect on HS dendrite scaling. (A)** Cartoon depicting parent–daughter scaling. Radii at branch points scale according to $r_{0}^{2}=r_{1}^{2}+r_{2}^{2}$, where $r_{0}$ is the radius of the parent branch, and $r_{1}$ and $r_{2}$ are the radii of the daughter branches. **(B)** Experimental measurements of parent–daughter scaling in control (open circles, left panel) and Milton KD (blue circles, right panel) dendrites, with $r_{0}^{2}$ plotted versus $r_{1}^{2}+r_{2}^{2}$. **(C)** Cartoon depicting sister–sister scaling. Sister subtrees scale such that subtree length is proportional to subtree volume and trunk thickness ($r^{2}$) is proportional to subtree bushiness (length/depth). **(D–E)** Experimental measurements of sister–sister scaling, with subtree length asymmetry plotted versus volume asymmetry (D) and trunk cross-sectional area asymmetry plotted versus subtree bushiness asymmetry (E). *N* = 438 branch points from 10 dendrites (control) and 256 branch points from 11 dendrites (Milton KD).

Thus far, our results demonstrate that mitochondria in fully mature HS dendrites are dynamic: they exhibit bidirectional transport throughout the lifetime of the neuron, and they fuse and exchange their contents. Next, we wished to determine how deficits in mitochondrial transport or fusion, induced during development, affect mitochondrial localization, dendrite morphology, and dendrite function. Mitochondrial transport in neurons is mediated by an adaptor protein, Milton (TRAK1/2 in vertebrates), that links the mitochondrial outer membrane protein Miro to kinesin and dynein (Fig. 3 A) (Glater et al., 2006; Stowers et al., 2002). We knocked down or overexpressed Milton in HS neurons during development and adulthood (using the R27B03-GAL4 driver, Fig. S1) and measured the effects in young flies (7 days old). Milton overexpression had no effect on mitochondrial transport (Fig. 3 C). In contrast, Milton KD abolished mitochondrial transport in HS primary dendrites, reducing linear flux in the anterograde and retrograde directions to zero (Fig. 3 B). Consistent with its effect on mitochondrial transport, Milton KD caused mitochondrial mislocalization, with mitochondria enriched in the soma and depleted from the distal dendrites (Fig. 3, D and E). Milton KD also caused a significant reduction in HS dendrite size (i.e., reducing the total volume, length, and number of branches per dendrite, Fig. 3, F and I) without affecting dendrite branch scaling (i.e., the relative widths of parent and daughter branches at each branch point, Fig. S3) (Donovan et al., 2024) or the total number of neurons per optic lobe (three neurons/optic lobe for all control and Milton KD samples, *N* = 9–10 optic lobes per genotype). Finally, Milton KD abolished calcium and ATP responses to PD and ND global motion (Fig. 3, J–K). Altogether, inhibition of mitochondrial transport, beginning prior to eclosion and continuing throughout adulthood, results in mitochondrial mislocalization and significant defects in HS dendrite form and function.

**Figure 4.**
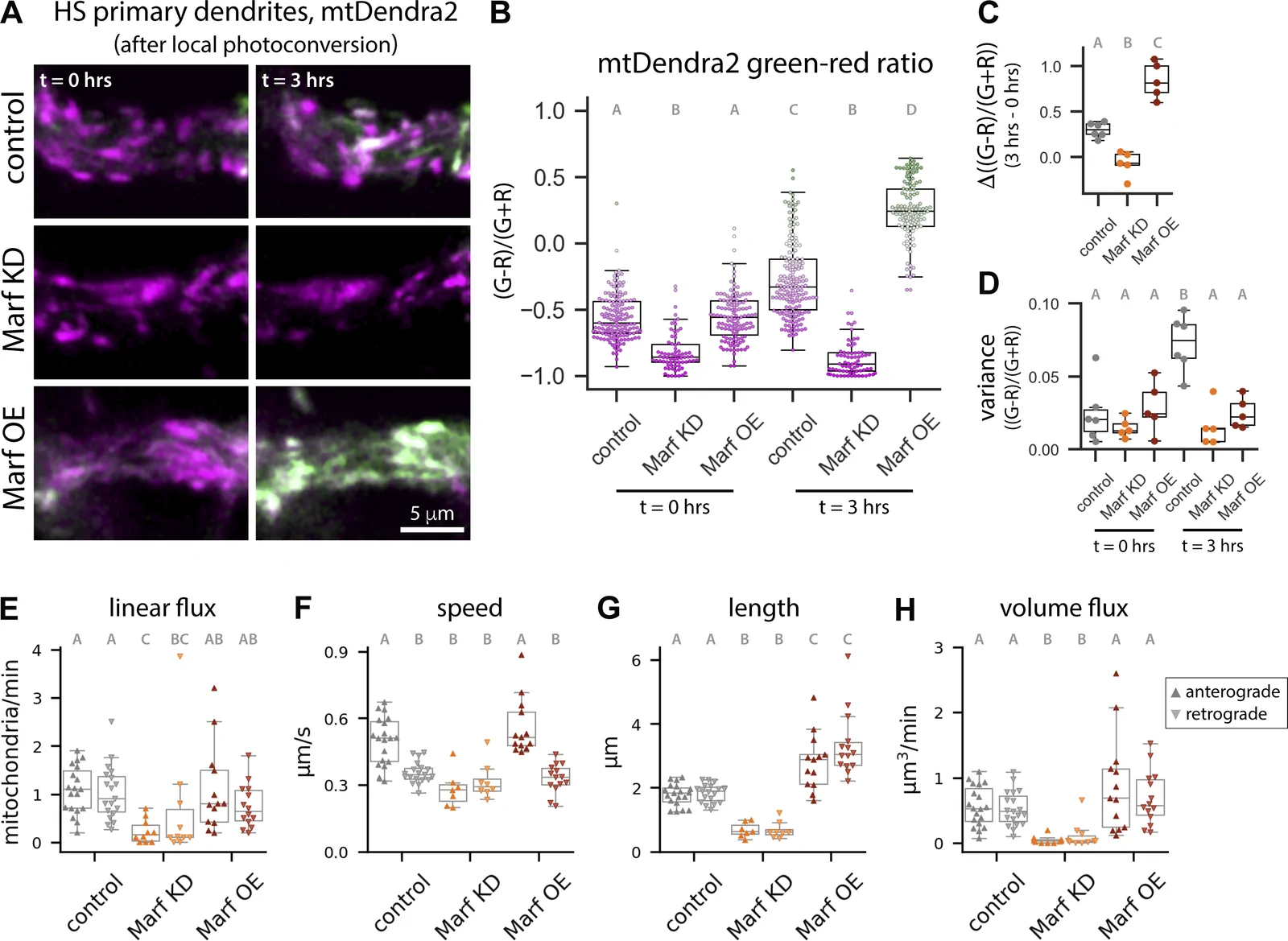
**Marf is necessary for mitochondrial transport and content exchange. (A)** *In vivo* confocal images of Dendra2-tagged mitochondria in primary HS dendrites immediately after photoconversion (t = 0 h) and 3 h later (t = 3 h) in control (top row), Marf KD (middle), and Marf OE (bottom) samples. **(B)** Green-to-red ratio in segmented mitochondria after photoconversion. Dots overlaid on the boxplots indicate measurements from individual mitochondria, and dot color reflects the green-to-red ratio within each mitochondrion. Letters indicate groups that are significantly different (Kruskal–Wallis test with post hoc Dunn’s test, P < 10^−8^). **(C and D)** Change in the average green-to-red ratio over time (3 h–0 h after photoconversion) for the population of mitochondria within a primary HS dendrite (C), and the variation in the green-to-red ratio across the population at 0 and 3 h after photoconversion (D). Dots overlaid on the box plots indicate measurements from individual neurons; *N* = 5–6 neurons per genotype. Letters indicate groups that are significantly different (ANOVA with post hoc Tukey’s test, P < 0.05). **(E–H)** Quantification of mitochondrial motility. Box plots show linear flux rates (E), average pause-free speeds (F), mitochondrial lengths (G), and total volume of mitochondria transported through the primary dendrite per minute (H) for anterograde and retrograde transport; dots overlaid on the box plots indicate average values for individual neurons (*N* = 10–19 neurons per genotype). Letters indicate groups that are significantly different (Kruskal–Wallis test with post hoc Dunn’s test, P < 0.01). OE, overexpression.

Next, to determine how disrupting mitochondrial content exchange during development and adulthood affects HS dendrites, we manipulated mitochondrial fusion by overexpressing or knocking down (via RNAi) the mitochondrial GTPase that mediates outer mitochondrial membrane (OMM) fusion, Marf (Mfn2 in vertebrates). Marf/Mfn2 drives OMM fusion via homotypic dimerization on adjacent mitochondria, and subsequent fusion of the inner mitochondrial membrane, mediated by the GTPase Opa1, results in exchange of mitochondrial matrix proteins and metabolites (Chan, 2020). Using local conversion of mtDendra2 in HS dendrites in young flies (7 days old), we found that Marf overexpression increased mitochondrial content exchange; ratios of green-to-red mtDendra2 fluorescence within mitochondria in the photoconverted ROI were significantly higher and less variable than in control samples 3 h after photoconversion (Fig. 4, A–D). In Marf KD samples, in contrast, photoconverted mitochondria remained entirely red 3 h after photoconversion (Fig. 4, A–D), indicating that Marf is necessary for mitochondrial content exchange.

In the absence of mitochondrial fusion, mitochondrial replenishment could in principle occur via transport-dependent mitochondrial replacement, with new mitochondria moving into the dendrite to replace old mitochondria. In our mtDendra2 experiments, we observed a small number of stationary green mitochondria in the photoconverted region 3 h after green-to-red photoconversion in control samples (Fig. 2 J; and Fig. 4, A and B). In Marf KD samples, however, no green mitochondria arrived in the photoconverted region (Fig. 4, A and B), suggesting that Marf plays a role in mitochondrial transport, as well as mitochondrial fusion. Consistent with this idea, knockout of the vertebrate homolog of Marf (Mfn2) reduced axonal transport of mitochondria in cultured neurons (Misko et al., 2010). Mfn2 has also been shown to immunoprecipitate with the vertebrate homologs of Milton and Miro (Misko et al., 2010), suggesting that Mfn2 regulates mitochondrial transport via direct interaction with transport machinery. To measure the effects of Marf manipulations on mitochondrial transport in HS dendrites, we imaged GFP-tagged mitochondria moving through primary dendrites. On average, motile mitochondria in control samples were ∼2 μm long (Fig. 2 G and Fig. 4 G). Marf KD significantly reduced the size of motile mitochondria (average length ∼0.7 μm), and Marf overexpression had the opposite effect (average length ∼3 μm) (Fig. 4 G). Marf overexpression had no effect on mitochondrial transport rates, but Marf KD significantly reduced the speed of mitochondria moving in the anterograde direction, and significantly reduced linear flux rates in both directions (Fig. 4, E and F). Together, the reduction in the size and number of motile mitochondria upon Marf KD dramatically reduced the total volume of mitochondria exchanged through the primary dendrite per minute (Fig. 4 H). These results indicate that Marf is necessary for mitochondrial transport, as well as mitochondrial content exchange in HS dendrites.

**Figure 5.**
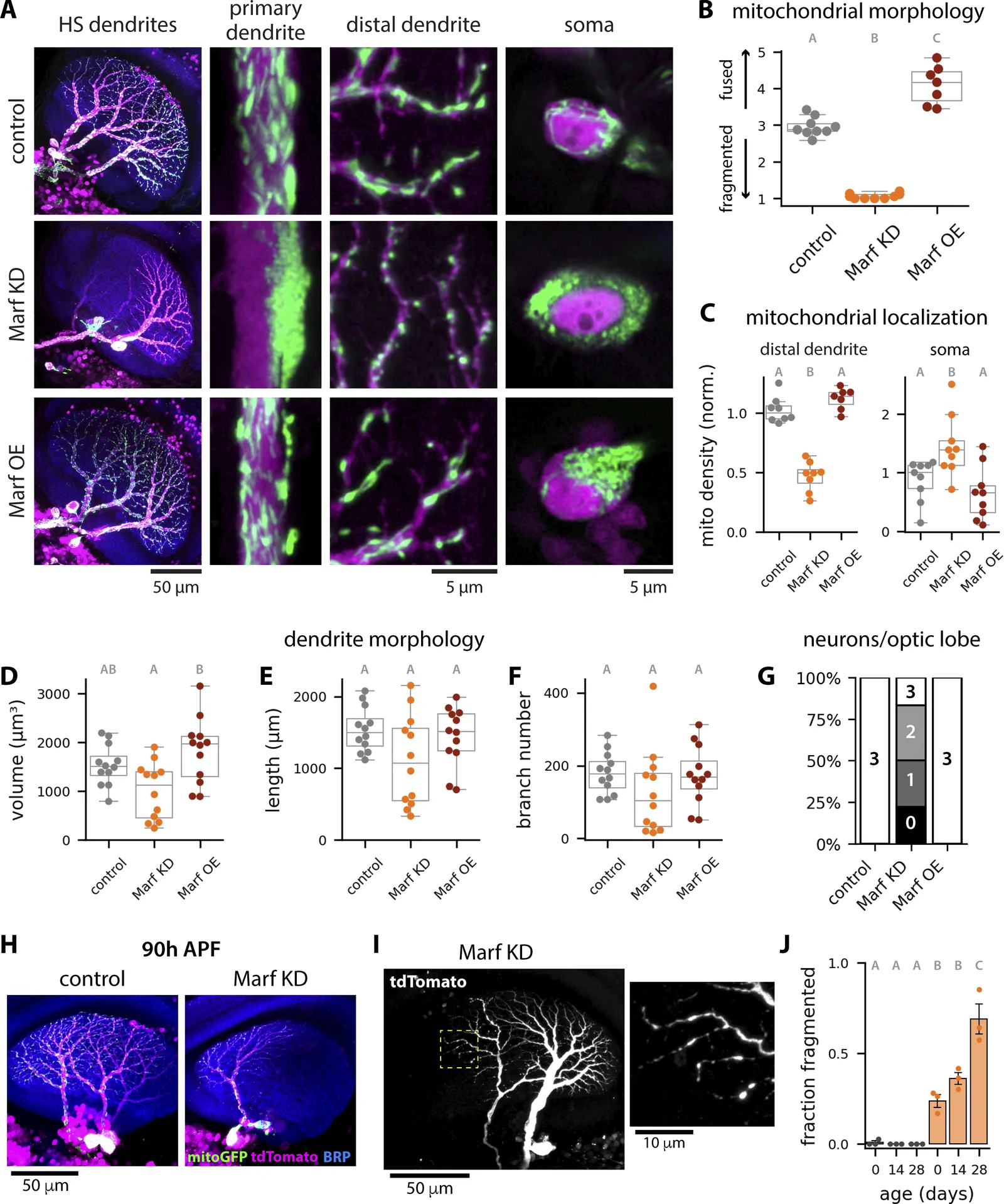
**Marf KD during development and adulthood causes mitochondrial fragmentation and mislocalization, reduced cell numbers, and age-dependent dendrite degeneration. (A)** Representative images of HS dendrites (tdTomato, magenta) and the mitochondria within them (mitoGFP, green) in control (top row), Marf KD (middle), and Marf OE (bottom) samples. **(B)** Mitochondrial morphology—fragmented versus fused—in HS distal dendrites. Dots overlaid on the box plots indicate measurements from individual flies (*N* = 7–9 flies per genotype). Letters indicate groups that are significantly different (Kruskal–Wallis test with post hoc Dunn’s test, P < 0.05). **(C)** Mitochondrial volume densities in distal dendrites (left) and somas (right). Dots overlaid on the box plots indicate average values for individual flies in the distal dendrites (*N* = 7–8 flies per genotype) or individual somas (*N* = 9 somas per genotype). Letters indicate groups that are significantly different (Kruskal–Wallis test with post hoc Dunn’s test, P < 0.01). **(D–F)** Dendrite volume (D), length (E), and total number of branches (F). Dots overlaid on the box plots indicate individual neurons (*N* = 12 neurons from 4 to 6 optic lobes). Letters indicate groups that are significantly different (Kruskal–Wallis test with post hoc Dunn’s test, P < 0.05). **(G)** Stacked bar plot showing the number of HS neurons per optic lobe in 7-day-old flies; *N* = 14–19 optic lobes per genotype. **(H)** Representative images of HS dendrites in control and Marf KD samples at 90 h APF, ∼10 h prior to eclosion. **(I)** Representative image of HS dendrites from a 14-day-old Marf KD sample; yellow dashed box indicates the region enlarged in the image on the right. **(J)** Fraction of HS dendrites that are degenerated, plotted as a function of age. Dots overlaid on the bar plot indicate the fraction fragmented per replicate (*N* = 3 replicates; five or more optic lobes per replicate). Letters indicate groups that are significantly different from each other (ANOVA with post hoc Tukey’s test, P < 0.05). APF, after pupal formation; OE, overexpression.

**Figure S4.**
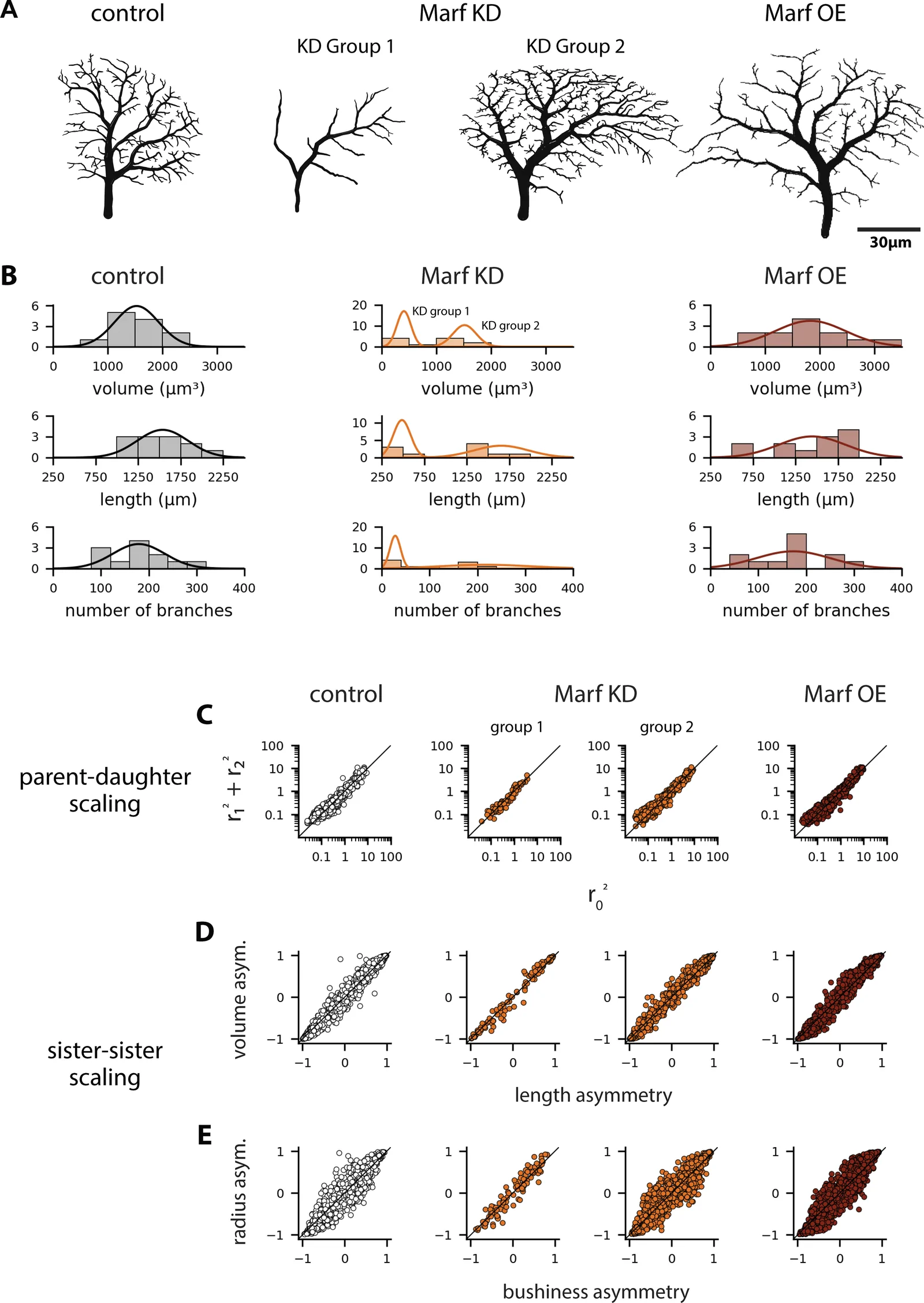
**Marf KD has a variable effect on HS dendrite size. (A)** Representative reconstructions of HS dendrites from control, Marf KD, and Marf OE samples. The two Marf KD dendrites illustrate the range of dendrite morphologies in the KD samples. **(B)** Histograms showing distributions of dendrite volumes, lengths, and number of branches for control (gray), Marf KD (orange), and Marf OE (maroon) samples. Gaussian curves overlaid on the histograms were defined by Gaussian mixture models; control and Marf OE distributions were best-fit by a single Gaussian, whereas Marf KD distributions were best-fit by two Gaussians. **(C)** Parent–daughter scaling in control (open circles, left panel), Marf KD (orange, center panels), and Marf OE (maroon, right panel) dendrites, with $r_{0}^{2}$ plotted versus $r_{1}^{2}+r_{2}^{2}$. **(D and E)** Sister–sister scaling, with subtree length asymmetry plotted versus volume asymmetry (D) and trunk cross-sectional area asymmetry plotted versus subtree bushiness asymmetry (E). Marf KD samples were divided into two groups based on the bimodal distributions shown in B. *N* = 1,564 branch points from 12 dendrites (control), 101 branch points from 5 dendrites (Marf KD group 1), 970 branch points from 6 dendrites (Marf KD group 2), and 1,502 branch points from 12 dendrite (Marf OE). OE, overexpression.

Having demonstrated that Marf is necessary for mitochondrial transport and content exchange, we next measured the effects of Marf KD on mitochondrial localization and dendrite architecture. Consistent with Marf’s role in OMM fusion, Marf KD resulted in mitochondrial fragmentation, and Marf overexpression caused mitochondrial elongation (Fig. 5, A and B). Similar to Milton KD, Marf KD also disrupted mitochondrial localization, reducing the mitochondrial volume density in the distal dendrites while increasing the mitochondrial density in the soma (Fig. 5 C). In addition, we noted that Marf KD resulted in accumulation of fragmented mitochondria in the soma stalk, a thin projection connecting the soma to the primary dendrite (as is typical of *Drosophila* neurons) (Fig. 5 A). Also similar to Milton KD, Marf KD reduced HS dendrite complexity, albeit in a more variable fashion, without affecting dendritic branch scaling (Fig. 5, D–F and Fig. S4). We measured bimodal distributions of dendrite volume, length, and number of branches; approximately half of the dendrites in our Marf KD samples were smaller than control dendrites, whereas the other half were indistinguishable from controls (Fig. S4). This bimodal distribution of dendrite sizes suggests that variable Marf KD caused a threshold effect, in which stronger (or earlier) KD reduced dendrite size in 7-day-old flies, but weaker (or later) KD did not.

Finally, Marf KD also reduced the number of HS dendrites per optic lobe (Fig. 5 G). In control flies, we observed three HS neurons in all optic lobes (*N* = 19 optic lobes, SD = 0); in Marf KD samples, we observed 1.4 ± 1 neurons/optic lobe (*N* = 18 optic lobes) in 7-day-old flies. The effects of Marf KD on neuron number were apparent prior to eclosion (Fig. 5 H), and the number of cells did not significantly decrease with age (average cell count at day 28 = 1.1 ± 0.7 neurons/optic lobe, *N* = 22 optic lobes), suggesting the reduction in cell count is due to developmental effects. This reduction in cell count is consistent with recent evidence that mitochondrial fusion and fission regulate neurogenesis (Dubal et al., 2022; Iwata et al., 2020). In addition to reduced cell counts, we also observed severe dendrite degeneration, characterized by fragmentation of the dendrite, in a subset of Marf KD samples (Fig. 5 I). The fraction of fragmented neurons increased over time, from ∼20% within 24 h of eclosion to ∼70% at 28 days (Fig. 5 J). Thus, Marf KD causes a reduction in cell count prior to eclosion, as well as dendrite degeneration that increases with age.

Altogether, these results indicate that deficits in mitochondrial dynamics, when induced prior to eclosion, disrupt dendrite morphology and function.

### HS dendrite morphology, visual stimulus-driven ATP responses, and direction selectivity are robust to adult-onset deficits in mitostasis

**Figure 6.**
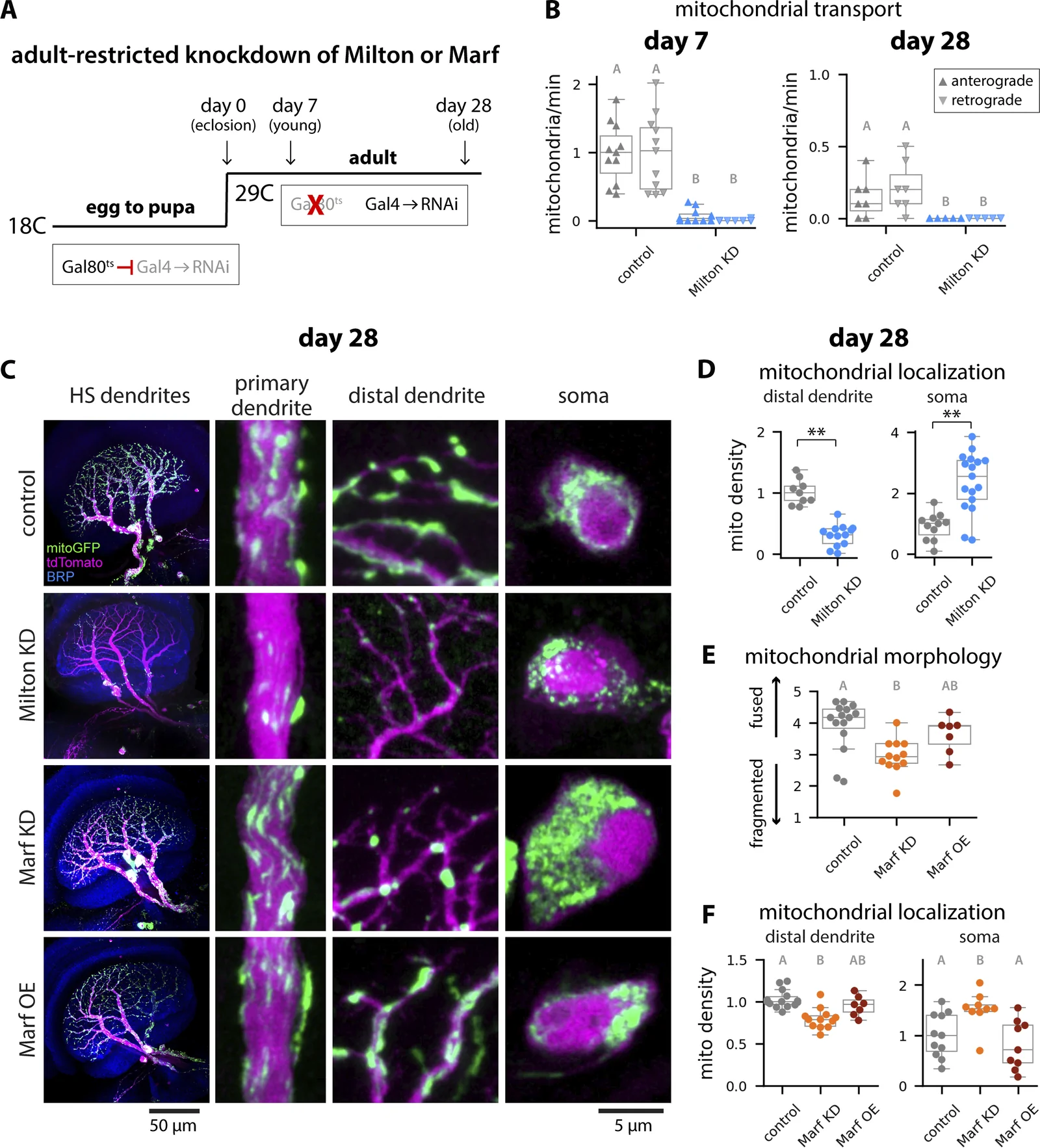
**Adult-restricted KD of Milton or Marf depletes mitochondria from HS dendrites. (A)** Experimental approach. Gal80^ts^ suppressed Gal4-driven expression in crosses reared at 18°C. After eclosion, adult flies were shifted to 29°C to inactivate Gal80^ts^ and allow shRNA expression in HS neurons. Experiments were conducted 7 or 28 days after the induction of shRNA expression. **(B)** Mitochondrial linear flux through primary HS dendrites in control and Milton KD samples at day 7 (left) and day 28 (right). Dots overlaid on the box plots indicate measurements from individual flies (*N* = 7–11 flies per genotype). Letters indicate groups that are significantly different (Kruskal–Wallis test with post hoc Dunn’s test, P < 0.001). **(C)** Representative images of HS dendrites (tdTomato, magenta) and the mitochondria within them (mitoGFP, green) in control, Milton KD, Marf KD, and Marf OE samples at day 28. Overview images on the left show all HS dendrites in the optic lobe; images of the primary and distal dendrites and somas are enlarged on the right. **(D)** Mitochondrial volume density in distal dendrites (left plot) and somas (right plot) at day 28 in control (gray) and Milton KD samples (blue). Dots overlaid on the box plots indicate average values for individual flies in the distal dendrites (*N* = 9–13 flies per genotype) or individual somas (*N* = 11–17 somas per genotype). Asterisks indicate significant differences (Mann–Whitney U test, P < 0.01). **(E and F)** Mitochondrial morphology—fragmented versus fused—in HS distal dendrites (E) and mitochondrial volume densities in distal dendrites and somas (F) at day 28 in control (gray, *N* = 14), Marf KD (orange, *N* = 12), and Marf OE (maroon, *N* = 7) samples. Letters indicate groups that are significantly different (Kruskal–Wallis test with post hoc Dunn’s test, P < 0.01). OE, overexpression.

**Figure S5.**
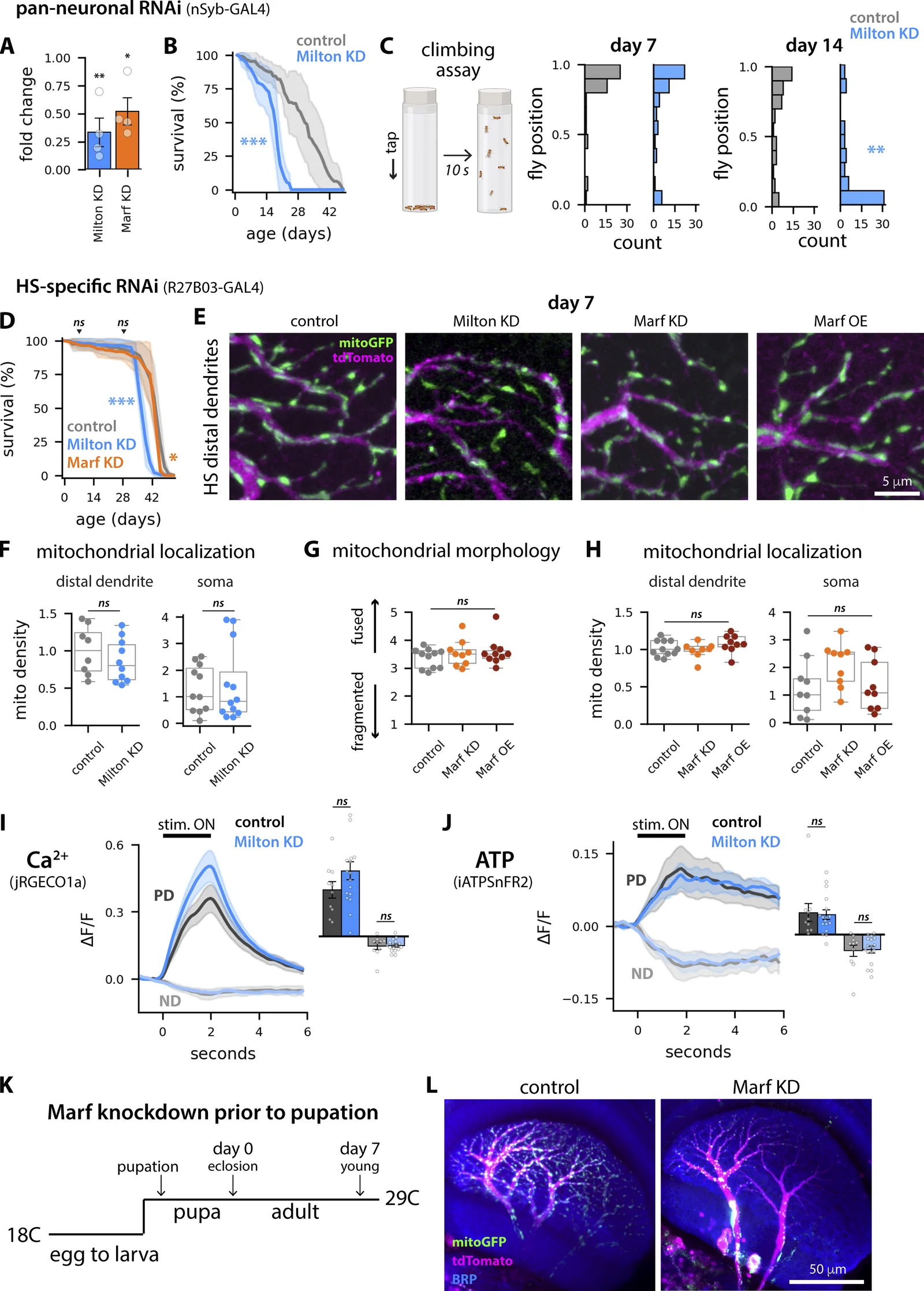
**Effects of adult-restricted perturbations of mitochondrial dynamics on fly longevity, locomotion, and HS dendrite function in young flies. (A)** Fold change in Milton and Marf transcript levels 7 days after pan-neuronal, adult-restricted (with Gal80^ts^) expression of shRNAi. Asterisks indicate significant differences from 1 (*P < 0.05, **P < 0.01, one-sample *t* test). **(B and C)** Effects of pan-neuronal, adult-restricted Milton KD on fly longevity (B) and climbing behavior (C). Asterisks indicate significant differences in longevity (B, log-rank test, ***P < 10^−10^) and median fly height in a climbing assay (C, Mann–Whitney U test, **P < 0.001). **(D)** Effects of HS-specific, adult-restricted Milton or Marf KD on fly longevity. Asterisks indicate a significant difference in lifespans of control versus Milton KD samples (log-rank test, *P < 0.001, ***P < 10^−10^); n.s. indicates no significant differences in survival rates at experimental time points (days 7 and 28, Mann–Whitney U test, P > 0.05). **(E)** Representative images of distal HS dendrites 7 days after HS-specific, adult-restricted induction of Milton or Marf KD or Marf overexpression. Neurons were labeled with mitoGFP (green) and tdTomato (magenta). Images are maximum projections of confocal z-stacks acquired using Airyscan imaging. **(F)** Mitochondrial volume densities, normalized to the median of the control, in distal dendrites (left panel) and somas (right panel) in control (gray) and Milton KD (blue) samples. Dots overlaid on the box plots indicate average measurements for individual flies in the distal dendrites (*N* = 8–9 flies per genotype) or measurements in individual somas (*N* = 11–12 somas per genotype). **(G)** Quantification of mitochondrial morphology in distal HS dendrites in control (gray), Marf KD (orange), and Marf overexpression (maroon) samples. Dots overlaid on boxplots indicate average measurements for individual flies (*N* = 9–11 flies per genotype). **(H)** Normalized mitochondrial volume densities in distal dendrites (left panel) and somas (right panel). Dots overlaid on the boxplots indicate average measurements for individual flies in the distal dendrites (*N* = 8–11 flies per genotype) and measurements in individual somas (*N* = 9 somas per genotype). **(I and J)** Average calcium (jRGECO1a, I) and ATP (iATPSnFR2, J) responses to wide-field square-wave gratings moving in the PD or ND in distal dendrites of control (gray) and Milton KD (blue) samples at day 7. Insets show average response amplitudes. Shading on the line plots and error bars on the bar plots indicate the standard error of the mean; dots overlaid on the bar plots are average values for individual flies (*N* = 10–16 flies per genotype). There are no significant differences among sample groups (Mann–Whitney U test, P > 0.05). **(K and L)** Experimental time course (K) and representative images (L) showing the effects of HS-specific Milton KD, induced prior to pupation, on mitochondrial localization and morphology.

Deficits in mitochondrial transport and fusion could reduce dendrite complexity in young adult flies due to developmental defects (e.g., reduced dendrite growth), loss of mitostasis in the adult and subsequent dendrite degeneration, or both. To determine whether adult-onset defects in mitochondrial dynamics disrupt dendrite maintenance, we used Gal80^ts^ to restrict Gal4-driven expression to adulthood (Fig. 6 A). We reared crosses at 18°C to suppress the expression of Milton or Marf RNAi during development, and we induced KD during adulthood by shifting the temperature to 29°C after eclosion. Using a pan-neuronal driver (nSyb-GAL4), we confirmed that the adult-restricted expression of Milton and Marf RNAi resulted in significant reductions in Milton and Marf transcript levels in fly heads (Fig. S5 A). In addition, pan-neuronal, adult-restricted KD of Milton significantly reduced fly longevity (Fig. S5 B), with the average lifespan declining from 30 ± 5 days for control flies to 17 ± 2 days for Milton KD flies. Pan-neuronal Milton KD also caused significant locomotor defects, which we quantified using a simple climbing assay (Fig. S5 C). Flies normally exhibit robust negative geotaxis behavior, rapidly climbing to the top of a vial after being tapped to the bottom (Madabattula et al., 2015). This climbing behavior was readily apparent in young (∼7 days old) pan-neuronal Milton KD flies, as well as controls: the majority of flies climbed to the top of the vial within a 10-s climbing period. In older flies, however (∼14 days old), pan-neuronal Milton KD resulted in significant defects in climbing behavior, with the majority of flies remaining at or near the bottom of the vial (Fig. S5 C). Thus, adult-restricted Milton KD in all neurons negatively impacts fly longevity and locomotion.

Next, we used an HS-specific driver (R27B03-GAL4), along with Gal80^ts^, to restrict RNAi expression to adult HS neurons (Fig. 6 A). Using this driver, adult-restricted Milton or Marf KD resulted in a small but significant decline in lifespan (average lifespan = 43 ± 8 [control], 37 ± 6 [Milton KD], and 41 ± 9 days [Marf KD]; Fig. S5 D), likely due to off-target expression in neurons in the central brain (Fig. S1). However, fly survival was unaffected by Milton or Marf KD at experimental time points (days 7 and 28). We measured near-complete inhibition of mitochondrial transport through primary dendrites at days 7 and 28 (Fig. 6 B). This inhibition of mitochondrial transport had no significant effect on mitochondrial localization at day 7 (Fig. S5, E and F), but mitochondria were depleted from the dendrite and retained in the soma over time, resulting in a significant loss of mitochondrial volume in the dendrite at day 28 (Fig. 6, C–D). Similarly, Marf KD had no effect on mitochondrial morphology or localization in young flies (7 days old) but reduced mitochondrial size in old flies (Fig. 6, C and E; and Fig. S5, E, G, and H). Marf KD also resulted in mitochondrial mislocalization over time, with mitochondria significantly depleted from the dendrites and enriched in the soma at day 28, albeit to a lesser extent than in Milton KD samples (Fig. 6 F). These results demonstrate that ongoing mitochondrial transport and fusion are necessary for mitostasis in adult HS dendrites. In addition, when restricted to the adult, we noticed that Marf KD had less dramatic effects on mitochondrial morphology and localization compared with constitutive Marf KD (i.e., throughout development and adulthood, Fig. 5. A–C). In principle, the relatively modest effect of adult-restricted Marf KD could be due to incomplete inactivation of Gal80^ts^ at the nonpermissive temperature, and thus weaker expression of Marf RNAi, or due to the timing of the KD (i.e., during adulthood only). To discriminate between these possibilities, we shifted to the nonpermissive temperature for Gal80^ts^ (29°C) approximately 4 days before eclosion, during the final larval stage (L3), in order to induce Marf RNAi expression in HS neurons during development (Fig. S5 K). Shifting to 29°C at L3 resulted in dramatic mitochondrial fragmentation and depletion from the dendrites in 7-day-old flies (Fig. S5 L), suggesting that adult-restricted Marf KD has a smaller effect on mitochondrial morphology and localization, compared with constitutive KD, due to the timing of the KD rather than incomplete inactivation of Gal80^ts^.

**Figure 7.**
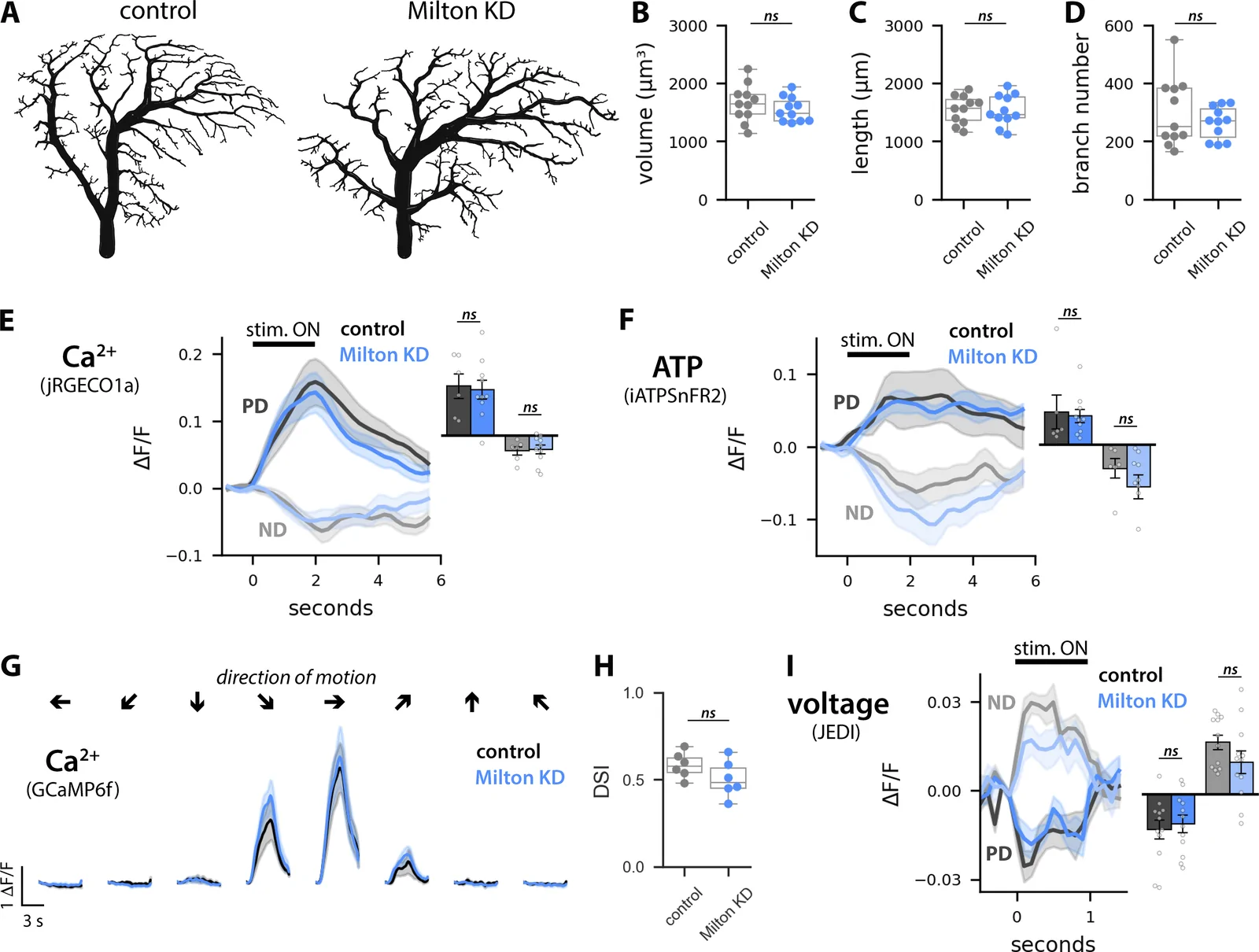
**Adult-onset Milton KD does not affect HS dendrite morphology or function. (A)** Representative reconstructions of control (left) and Milton KD (right) HS dendrites in 28-day-old flies. **(B–D)** Dendrite volume (B), length (C), and number of branch points (D) in control (gray) and Milton KD (blue) samples at day 28. Dots overlaid on the box plots indicate measurements for individual dendrites (*N* = 11 dendrites per genotype). **(E and F)** Average calcium (jRGECO1a, E) and ATP (iATPSnFR2, F) responses to wide-field square-wave gratings moving in the PD or ND in distal dendrites of control (gray) and Milton KD (blue) samples at day 28. Insets show average response amplitudes. Shading on the line plots and error bars on the bar plots indicate the standard error of the mean; dots overlaid on the bar plots are average values for individual flies (*N* = 6–10 flies per genotype). **(G and H)** Average dendritic calcium (GCaMP6f) responses (G) and DSI (H) for wide-field square-wave gratings moving in eight different directions for control (gray) and Milton KD (blue) samples at day 28. Dots overlaid on the box plot indicate measurements for individual flies (*N* = 6 flies per genotype). **(I)** Average voltage (JEDI) responses to wide-field square-wave gratings moving in the PD or ND in distal dendrites of control (gray) and Milton KD (blue) samples at day 28. Inset shows average response amplitudes; *N* = 12 flies per genotype. There are no significant differences between control and Milton KD samples (Mann–Whitney U test, P > 0.05).

**Figure 8.**
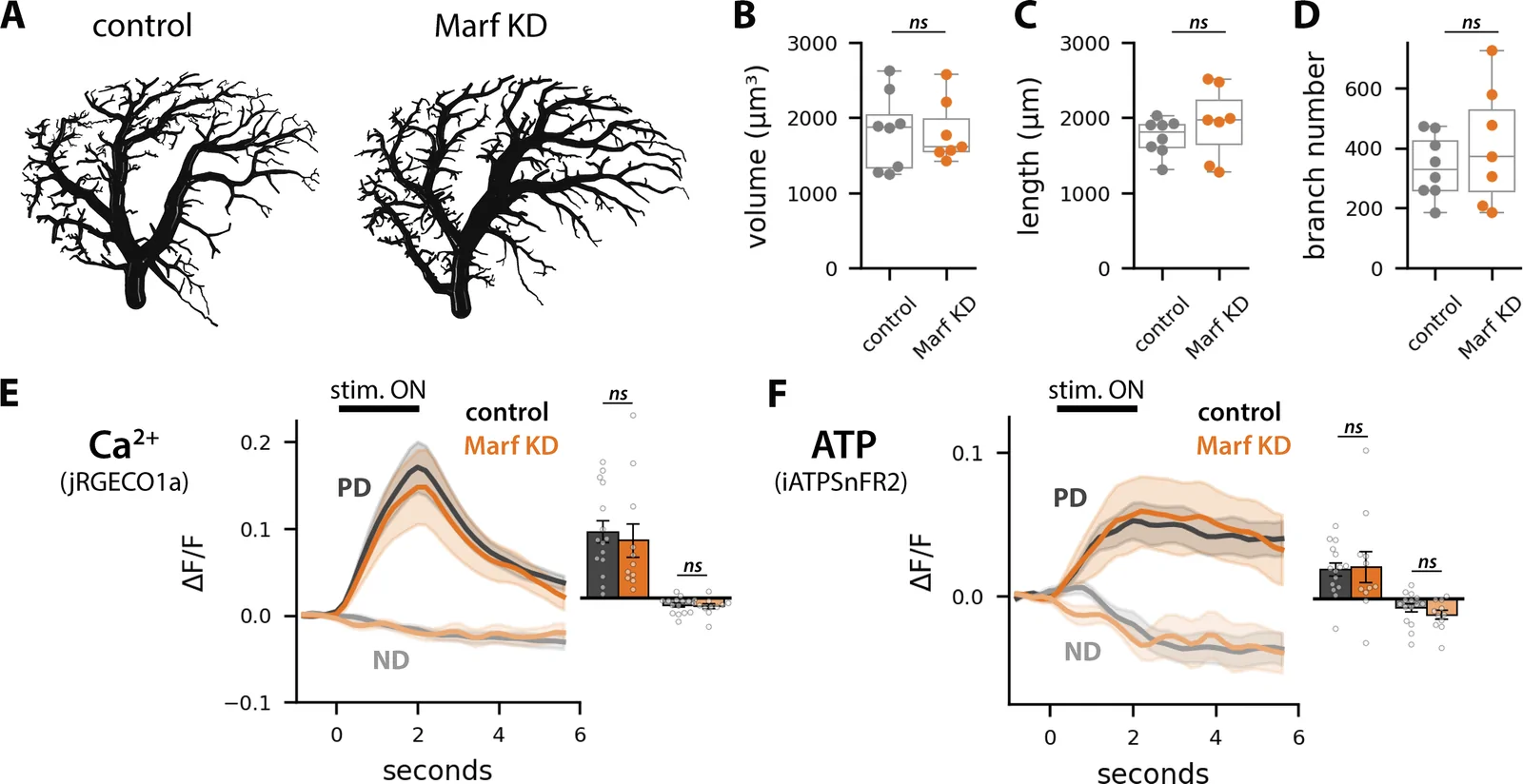
**Adult-onset Marf KD does not affect HS dendrite morphology or visual stimulus-driven ATP responses. (A)** Representative reconstructions of control (left) and Marf KD (right) HS dendrites in 28-day-old flies. **(B–D)** Dendrite volume (B), length (C), and number of branch points (D) in control (gray) and Marf KD (orange) samples at day 28 (*N* = 7–8 dendrites per genotype). **(E and F)** Average calcium (jRGECO1a, E) and ATP (iATPSnFR2, F) responses to wide-field square-wave gratings moving in the PD or ND in distal dendrites of control (gray) and Marf KD (orange) samples (*N* = 11–15 flies per genotype). There are no significant differences between control and Marf KD samples (Mann–Whitney U test, P > 0.05).

Having found that adult-restricted KD of Milton or Marf disrupts mitostasis in mature HS dendrites, we next examined the effects of these manipulations on the maintenance of the dendrite. Interestingly, neither Milton nor Marf adult-restricted KD affected the number or morphology of HS dendrites in 28-day-old flies (Fig. 7, A–D and Fig. 8, A–D), despite causing significant mitochondrial depletion (Fig. 6, C, D, and F). We observed three HS neurons per optic lobe for all samples (*N* = 7–11 optic lobes per genotype), and neither Milton nor Marf KD, when restricted to the adult, had an effect on dendrite volume, length, or branch number (Fig. 7, B–D and Fig. 8, B–D). Thus, in fully developed HS neurons, dendrite morphology is robust to Milton and Marf KD, suggesting the reduced dendrite complexity and age-dependent dendrite degeneration we observed upon KD prior to eclosion (Figs. 3 and 5) are due to deficits established during development.

Next, to determine how adult-restricted Milton or Marf KD affect HS dendrite function, we used *in vivo* two-photon imaging of head-fixed flies. To measure stimulus-driven calcium and ATP responses, we presented full-contrast square-wave gratings, moving in either the PD or the ND, on a screen positioned in front of the fly while simultaneously imaging jRGECO1a and iATPSnFR2 in distal HS dendrites. Adult-restricted Milton KD had no effect on mitochondrial localization in young flies (Fig. S5, E and F), and we were therefore unsurprised to find it had no effect on calcium or ATP responses at the same time point: PD motion drove increases in calcium and ATP levels, whereas ND motion had the opposite effect, in both control and Milton KD 7-day-old samples (Fig. S5, I and J). We were surprised, however, to find that although Milton KD depleted mitochondria from the dendrite in old flies (Fig. 6, C and D), this mitochondrial depletion had no effect on calcium or ATP responses (Fig. 7, E and F). In control dendrites, calcium response amplitudes declined with age (Fig. 7 E and Fig. S5 I), and Milton KD neither exacerbated nor ameliorated this age-related decline: stimulus-driven calcium signals in 28-day-old Milton KD samples were indistinguishable from controls (Fig. 7 E). Milton KD also had no effect on PD motion-driven increases in ATP levels (Fig. 7 F), suggesting that depletion of mitochondria does not negatively impact the dendrite’s ability to upregulate ATP production in response to excitatory input. In addition, although ND motion drove slightly larger reductions in ATP levels in Milton KD samples compared with controls, suggesting that mitochondrial depletion from the distal dendrite may cause ATP consumption to further outpace production during stimulation, this difference was not statistically significant (Fig. 7 F). Adult-restricted Marf KD also had no measurable effect on stimulus-driven calcium or ATP responses in 28-day-old flies (Fig. 8, E and F), despite causing significant mitochondrial mislocalization and fragmentation at the same time point (Fig. 6, C, E, and F). Altogether, these results demonstrate that in HS dendrites, visual stimulus-driven fluctuations in ATP are largely impervious to adult-onset deficits in mitochondrial dynamics and localization.

HS dendrites have a well-defined function: to selectively detect global optic flow in a specific direction (front-to-back, with respect to the fly’s eye). In principle, it is possible that adult-onset inhibition of mitochondrial dynamics, and the resulting depletion of mitochondria from the dendrite, affects mitochondria-related functions other than ATP production (e.g., calcium buffering, redox balance, ROS production), which, in turn, negatively impacts HS function (i.e., direction selectivity). To examine the effects of mitochondrial depletion (via Milton KD) on HS dendrite direction selectivity, we used a high-affinity, genetically encoded calcium reporter (GCaMP6f) to measure dendritic calcium responses to wide-field square-wave gratings moving in eight different directions (Fig. 7 G). A narrow range of directions (0° ± 45°, where 0° is the PD) drove dendritic calcium responses (i.e., increased GCaMP6f fluorescence) in both control and Milton KD dendrites, and Milton KD had no significant effect on response amplitudes for any direction of motion (Fig. 7 G). To measure direction selectivity, we calculated a direction selectivity index (DSI) by dividing the amplitude of the response to PD motion by the sum of the responses to all directions. Milton KD had no significant effect on the direction selectivity of dendritic calcium responses (Fig. 7 H). In addition, previously published patch-clamp recordings from the soma have shown that HS voltage responses to global motion are also direction-selective: HS neurons depolarize in response to PD motion and hyperpolarize in response to ND motion (Schnell et al., 2010). To measure the effect of Milton KD on voltage responses to PD and ND motion in the dendrite, we used a genetically encoded voltage indicator (JEDI-2P [Liu et al., 2022]). We found that JEDI fluorescence intensity decreased (indicating depolarization) in response to PD motion and increased (indicating hyperpolarization) in response to ND motion in both control and Milton KD, and Milton KD had no significant effect on the amplitude of the response to either direction of motion (Fig. 7 I). Thus, adult-restricted Milton KD has no measurable effect on the direction selectivity of dendritic calcium or voltage responses.

**Figure 9.**
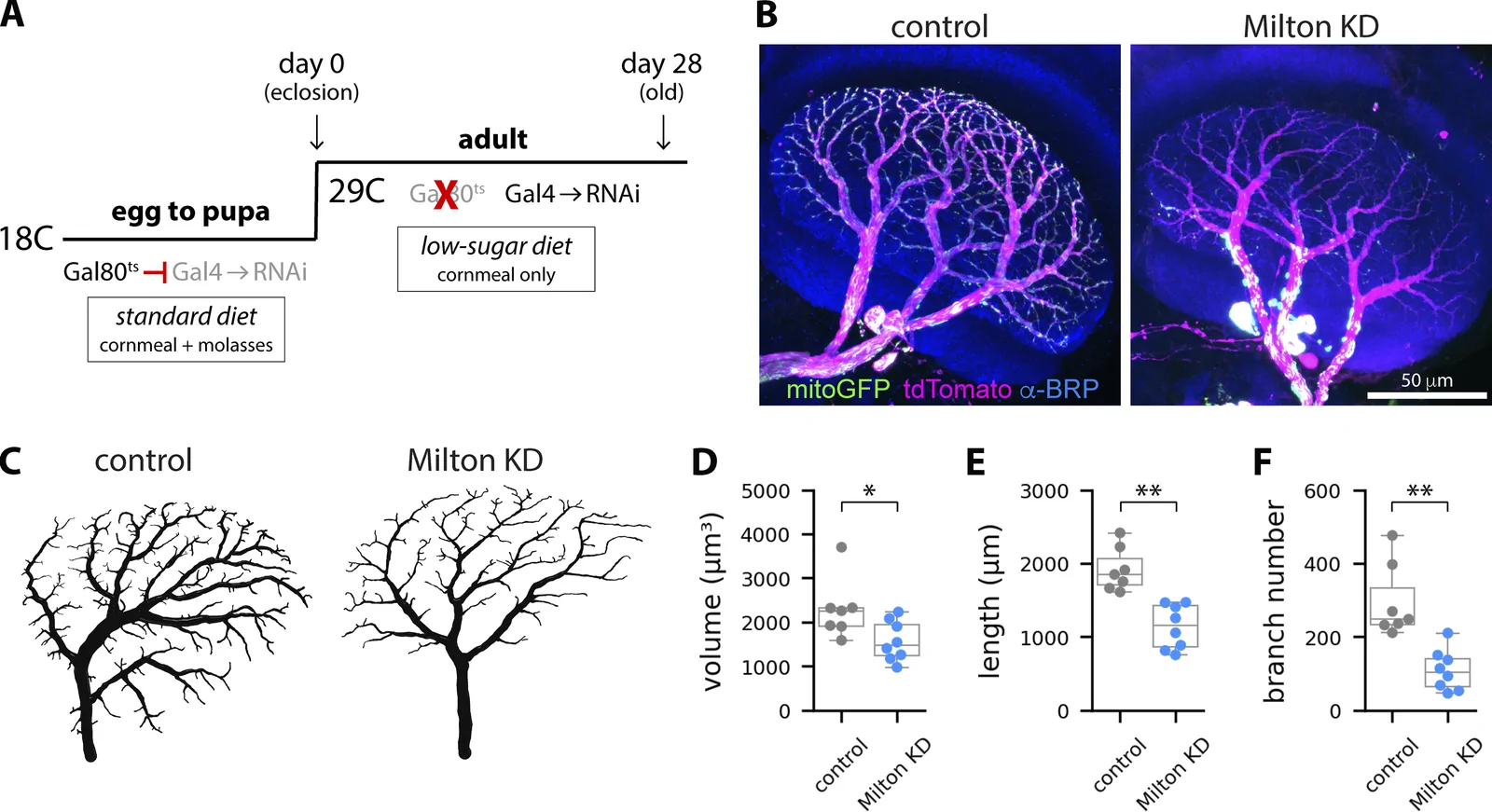
**Low-sugar diet sensitizes HS dendrites to mitochondrial depletion. (A)** Schematic depicting experimental time course for combining adult-restricted Milton KD with a low-sugar diet. After eclosion, adult flies were shifted to 29°C to inactivate Gal80^ts^ and allow Milton shRNA expression in HS neurons; at the same time, flies were shifted from standard cornmeal molasses food to a low-sugar diet (cornmeal only). Experiments were conducted 28 days after eclosion. **(B and C)** Representative images (B) and reconstructions (C) of HS dendrites in control (right) and Milton KD (left) samples. **(D–F)** Dendrite volume (D), length (E), and number of branch points (F) in control (gray) and Milton KD (blue) samples. Dots overlaid on the boxplots are measurements from individual dendrites (*N* = 7–8 dendrites per genotype); asterisks indicate significant differences (Mann–Whitney U test; *P < 0.05, **P < 0.001).

Finally, our results demonstrate that under physiological conditions *in vivo*, HS dendrites are remarkably resilient to adult-onset deficits in mitochondrial dynamics, even upon transport inhibition and significant depletion of mitochondria from the dendrite (Figs. 6, 7, and 8). Dendrite resilience to mitochondrial depletion suggests that alternative power management mechanisms may be sufficient to coordinate ATP supply with demand and maintain dendrite form and function. In particular, abundant evidence suggests that neurons dynamically regulate glycolysis to meet energetic demands (Ashrafi et al., 2017; Jang et al., 2016; Rangaraju et al., 2014; Richhariya et al., 2025; Wolfe et al., 2024). We reasoned that if glycolysis supports HS dendrites when mitochondria are depleted, then reducing glucose availability ought to sensitize the dendrites to adult-onset transport inhibition and mitochondrial depletion. To test this idea, we combined adult-restricted Milton KD in HS neurons (using Gal80^ts^ as described above) with dietary restriction of sugar. We reared crosses at 18°C on standard cornmeal molasses food, moved adult flies onto a low-sugar diet (cornmeal only) at 29°C to induce Milton KD immediately after eclosion, and measured HS dendrite morphology in 28-day-old flies (Fig. 9 A). In control flies kept on the low-sugar diet, HS dendrites were large and elaborately branched (Fig. 9, B–F), comparable to dendrites in flies reared on standard food (Fig. 7, A–D). In contrast, when combined with adult-restricted Milton KD, sugar restriction reduced HS dendrite complexity: dendrite volume, length, and number of branches were significantly lower than in control dendrites (Fig. 9, B–F). Taken together, our results suggest that HS dendrites are resilient to either mitochondrial depletion (upon transport inhibition) or reduced glycolysis (upon dietary restriction of sugar), but not both.

## Discussion

In this study, we found that *Drosophila* HS dendrites are surprisingly resilient to adult-onset deficits in mitochondrial dynamics. Whereas disruption of mitochondrial transport and content exchange during development caused a range of significant phenotypes—mitochondrial fragmentation and mislocalization, loss of dendrite complexity, reduced cell counts, age-related dendrite degeneration, and complete abrogation of visual stimulus-driven calcium and ATP fluctuations—adult-restricted perturbations depleted mitochondria from the dendrite without affecting dendrite morphology or stimulus-driven calcium, voltage, or ATP responses. Our results demonstrate that once properly developed, dendrites are capable of maintaining form and function *in vivo* upon mitochondrial depletion.

We show here that mitochondria in fully developed HS dendrites are highly dynamic, exhibiting substantial bidirectional transport and fusion-mediated content exchange. Moreover, we demonstrate that ongoing mitochondrial transport is necessary for mitostasis: adult-restricted inhibition of mitochondrial transport (via Milton KD) caused gradual depletion of mitochondria from distal HS dendrites, with mitochondrial volume density reduced to ∼30% of the normal density 4 weeks after induction of the KD. This timescale for mitochondrial depletion is roughly equivalent to the timescale for mitochondrial protein turnover in *Drosophila* (median mitochondrial protein half-life = ∼2 wk) (Vincow et al., 2013), suggesting that absent mitochondrial transport, basal rates of mitochondrial degradation result in local loss of mitochondrial mass over time. Moreover, although some previously published measurements suggest that old, depolarized mitochondria are selectively trafficked back to the soma for degradation (Lin et al., 2017; Miller and Sheetz, 2004), adult-restricted Milton KD abolished all mitochondrial transport, including retrograde transport to the soma. Distal depletion of mitochondria is therefore likely due to local degradation of mitochondria, either via piecemeal degradation of mitochondrial proteins and mitochondria-derived vesicles or via local mitophagy or macroautophagy followed by retrograde trafficking of autophagosomes (Ashrafi et al., 2014; Cason et al., 2022; Goldsmith et al., 2022; Maday et al., 2012; Misgeld and Schwarz, 2017).

Despite causing mitochondrial depletion from the dendrite, adult-restricted inhibition of mitochondrial transport or fusion had no effect on HS dendrite morphology, stimulus-driven ATP fluctuations, or direction selectivity under normal fly-rearing conditions (Fig. 6, 7, and 8), indicating that tight control of mitochondrial density is not strictly necessary for neuronal maintenance or function *in vivo*. In mice, some neuronal cell types can also maintain normal morphologies for extended periods of time upon mitochondrial depletion (Berthet et al., 2014; López-Doménech et al., 2016). In cortical pyramidal neurons, postnatal inhibition of mitochondrial transport (via knockout of Miro1) caused significant depletion of mitochondria from dendrites by 4 months of age, with a sizable fraction of dendritic branches entirely devoid of mitochondria (López-Doménech et al., 2016). Despite this mitochondrial depletion, dendrite morphologies were indistinguishable from controls at 4 months. Morphological defects did eventually appear—reduced dendrite branching and neuronal degeneration occurred by 8 and 12 months of age, respectively—but the delayed onset of these defects, relative to the timing of mitochondrial depletion, indicates that pyramidal neurons are able to maintain normal dendrite morphology in the absence of mitochondria for a surprisingly long time (months). In addition to depending on age, the consequences of mitochondrial depletion also appear to depend on neuronal cell type. In dopaminergic (DA) neurons, postnatal knockout of the fission factor Drp1 depleted mitochondria from axons throughout the mouse midbrain but only caused degeneration in a subset of DA neurons, with susceptible neurons enriched in the substantia nigra and resilient neurons enriched in the ventral tegmental area (Berthet et al., 2014). In Purkinje cells, postnatal knockout of Mfn2 caused mitochondrial mislocalization and dendrite degeneration by 2 months of age and cell death by 6 months (Chen et al., 2007). The idea that susceptibility to mitochondrial perturbations varies with neuronal cell type is consistent with neurodegenerative disease phenotypes. Heterozygous mutations in different fusion factors affect distinct populations of neurons: Mfn2 mutations are associated with axonopathy of peripheral motor and sensory neurons in Charcot–Marie–Tooth disease type 2A (Züchner et al., 2004), whereas Opa1 mutations are associated with progressive degeneration of retinal ganglion cells in dominant optic atrophy (Delettre et al., 2000). Similarly, mutations in master regulators of mitophagy, Pink1 and Parkin, cause selective degeneration of DA neurons in the substantia nigra in Parkinson’s disease (Pickrell and Youle, 2015). It remains unclear, however, why these mitochondrial mutations perturb specific subsets of neurons while other neuronal cell types are unaffected.

Neuronal susceptibility versus resilience to mitochondrial insults may be due to cell type–dependent differences in energetic demands (Alle et al., 2009; Carter and Bean, 2009; Niven, 2016). In addition, engagement of alternative power management mechanisms (e.g., dynamic regulation of glycolysis) could confer resilience to mitochondrial depletion or dysfunction in a cell type–specific fashion. In HS dendrites, adult-onset mitochondrial depletion had no significant effect on visual stimulus-driven increases in ATP, suggesting that HS dendrites do not rely on mitochondrial OXPHOS for local, acute regulation of ATP production. Instead, visual stimulus-driven fluctuations in ATP levels may be due to activity-dependent regulation of glycolytic flux. Consistent with this idea, blocking glycolysis causes a significant reduction in ATP levels upon neuronal activation in culture (Rangaraju et al., 2014). Moreover, recent work has shown that neurons in *Caenorhabditis elegans* tune glycolysis rates in a cell type–dependent fashion (Wolfe et al., 2024), as well as localizing glycolytic enzymes to synapses (Jang et al., 2016) and upregulating glycolytic flux under hypoxic conditions (Wolfe et al., 2024). In circadian clock neurons in *Drosophila*, cell type–specific knockout of either Marf or Opa1 triggers increased expression of glycolysis enzymes (Richhariya et al., 2025), suggesting that neurons can compensate for mitochondrial dysfunction via upregulation of glycolysis. Here, we show that a low-sugar diet sensitizes HS dendrites to mitochondrial depletion, resulting in a loss of dendrite complexity in Milton KD samples (Fig. 9). This result is consistent with the idea that glycolysis plays an important role in dendrite maintenance upon mitochondrial depletion, highlighting the need to determine how dynamic regulation of glycolysis contributes to neuronal function *in vivo*. In particular, future work is necessary to determine whether glycolysis contributes to the rapid, stimulus-locked ATP fluctuations we have observed in HS dendrites, as well as whether mitochondrial depletion induces compensatory upregulation of glycolysis in HS dendrites to maintain ATP homeostasis and thus dendrite form and function.

Moreover, in our experiments, depletion of mitochondria from the dendrite upon Milton or Marf KD is associated with enrichment of mitochondria in the soma. In this context, spatiotemporal energy buffering, mediated by metabolically inert phosphagens, may be particularly important to maintain baseline ATP levels in the dendrite. In phosphagen buffering systems, which have been extensively studied in muscle, creatine kinase (or arginine kinase, in flies) is functionally coupled with glycolysis or OXPHOS (Wallimann et al., 1992). At sites of high ATP production, creatine kinase catalyzes the transfer of the terminal phosphate group of ATP to creatine (or arginine), followed by diffusion of phosphocreatine throughout the cell. The resultant global pool of phosphocreatine, maintained in excess of ATP, allows creatine kinase to efficiently transfer the phosphate back to ADP at sites of high ATP consumption, thereby buffering fluctuations in ATP in space and time. In our experiments, inhibition of mitochondrial transport results in redistribution of mitochondria, with mitochondria enriched in the soma and depleted from the dendrite. This spatial distribution of mitochondria is reminiscent of mitochondrial localization in photoreceptors in salamanders and zebrafish, in which mitochondria are enriched in photoreceptor inner segments and excluded from synaptic terminals (Linton et al., 2010). In these photoreceptors, energy (in the form of phosphocreatine) flows from mitochondria toward synaptic terminals. In HS neurons, energetic flux from the soma to the distal dendrites, mediated by the *Drosophila* phosphagen system (arginine kinase/phosphoarginine), may protect the dendrite from degeneration when mitochondria are mislocalized.

Finally, mitochondria are normally densely distributed throughout HS dendrites, with mitochondria occupying ∼50% of the volume of distal dendrites (Donovan et al., 2024). Our measurements of mitochondrial transport and content exchange demonstrate that the mitochondrial network is also highly dynamic (Fig. 2), and it is surprising that mitochondrial depletion from mature HS dendrites has no effect on the primary function of the dendrite (i.e., direction-selective global motion detection, Fig. 7, G–I). Why would HS dendrites maintain such a large volume of dynamic mitochondria, which itself is likely to be energetically costly (Bergmann et al., 2025), if mitochondria are not strictly necessary to maintain dendritic function? Mitochondrial maintenance may ensure metabolic flexibility, allowing neurons to burn alternative fuel sources, including fatty acids (Kumar et al., 2025), when glucose is limited. In addition, if mitochondrial depletion or dysfunction triggers compensatory upregulation of glycolysis, as suggested by recent work in *Drosophila* circadian clock neurons (Richhariya et al., 2025), neurons that fail to maintain mitochondria may consume a disproportionately large share of the brain’s finite supply of glucose. Although mature HS dendrites are resilient to mitochondrial depletion under normal fly-rearing conditions, pan-neuronal Milton KD in adult flies results in severe locomotor defects and reduced lifespan (Fig. S5, B and C). Neurons may invest in mitochondrial maintenance, even if they are able to function without mitochondria *in vivo*, to prevent a tragedy of the commons (Hardin, 1968) in which escalating consumption of a finite fuel source by individual cells results in the collapse of energy homeostasis at the tissue level.

## Materials and methods

### *Drosophila* strains and husbandry

Flies were reared on standard molasses cornmeal food under a regular 12-h light/dark cycle at 25°C, unless otherwise indicated. To restrict the expression of transgenes to the adult using Gal80^ts^, crosses were reared at 18°C, and progeny were collected within a day of eclosion and moved to 29°C. To measure the effects of a low-sugar diet on dendrite morphology, crosses were reared on standard molasses cornmeal food and progeny collected at the day of eclosion were moved onto low-sugar food: 48.5 g/l cornmeal, 45.3 g/l yeast, 4.55 g/l agar, 0.5 g/l CaCl_2_, 0.5 g/l MgSO_4_. All crosses were flipped onto fresh food every 3–4 days. All *Drosophila* strains used in this work are listed in Table 1.

**Table 1.** Reagent or resource

Reagent or resource	Source	Identifier
**Antibodies**	​	​
Chicken polyclonal anti-GFP	Aves Labs	GFP-1010; RRID: AB_2307313
Rabbit polyclonal anti-RFP	Rockland	600-401-379; RRID: AB_2209751
Mouse monoclonal anti-BRP	DSHB	nc-82; RRID: AB_2314866
Alexa Fluor 488 goat anti-chicken IgY	Thermo Fisher Scientific	A-11039; RRID: AB_2534096
Alexa Fluor 555 goat anti-rabbit IgG	Thermo Fisher Scientific	A-21428; RRID: AB_2535849
Alexa Fluor 647 goat anti-mouse IgG	Thermo Fisher Scientific	A-21235; RRID: AB_2535804
**Chemicals**	​	​
Bondic UV glue	BONDIC	N/A
ProLong Gold Antifade Mountant	Invitrogen	S36937
PFA 16%	Electron Microscopy Sciences	15710
Triton X-100	Biomatik	A4025
TRIgent (TRIzol)	Biomatik	K5161
Chloroform	Sigma-Aldrich	288306
UltraPure DNase/RNase-Free Distilled Water	Thermo Fisher Scientific	10–977-015
2-Propanol	Sigma-Aldrich	190764
Ethanol	EMD Millipore	108543
RevertAid First Strand cDNA Synthesis Kit	Thermo Fisher Scientific	K1621
Invitrogen DNase I, Amplification Grade	Invitrogen	18-068-015
SYBR Green Universal Master Mix	Thermo Fisher Scientific	4309155
Custom 25 nmole DNA oligos (qRT-PCR primers)	IDT Technologies	N/A
**Experimental models: organisms/strains**	​	​
Canton-S	Bloomington *Drosophila* Stock Center (BDSC)	64349
GMR27B03-GAL4 (HS)	BDSC	49211
GMR57C10-GAL4 (nSyb)	BDSC	39171
w+; UAS-mitoGFP,UAS-tdTomato/CyO; GMR27B03-GAL4/TM6B	Donovan et al. (2024)	N/A
UAS-mtDendra2	Gift from Catherine Collins, Case Western University, Cleveland, OH, USA	N/A
UAS-mIFP-T2A-HO1	BDSC	64181
w+: UAS-mtDendra2/CyO; GMR27B03-GAL4, UAS-mIFP-T2A-HO1/TM6B	This study	N/A
UAS-jRGECO1a/TM6B	BDSC	63794
UAS-iATPSnFR2	Gift from Tom Clandinin, Stanford University, Stanford, CA, USA	N/A
w+; UAS-iATPSnFR2/CyO; GMR27B03-GAL4, UAS-jRGECO1a/TM6B	This study	N/A
UAS-pHerry	Gift from Gregory Macleod, Tulane University, New Orleans, LA, USA	N/A
UAS-GCaMP6f	BDSC	42747
UAS-JEDI-2P	BDSC	606048
UAS-Milton RNAi	BDSC	44477
UAS-Milton	BDSC	606820
UAS-Marf RNAi	BDSC	67158
UAS-Marf.HA/Cyo:TM6B	BDSC	67157
tubP-GAL80^ts^; TM2/TM6B	BDSC	7108
**Software and algorithms**	​	​
Fiji	​	fiji.sc
Ilastik	Berg et al. (2019)	www.ilastik.org
MATLAB	MathWorks	www.mathworks.com
PsychoPy	​	www.psychopy.org
Python3	Python	http://www.anaconda.com/products/distribution
R	​	https://www.r-project.org/
neuTube	Feng et al. (2015)	http://neutracing.com/
nnU-NET	Isensee et al. (2021)	http://github.com/mic-dkfz/nnunet
Custom-written Python code for quantifying mitochondrial transport, mitochondrial mixing, mitochondrial morphology, dendrite fragmentation, and extracting visual stimulus-driven calcium signals from *in vivo* images	Donovan et al. (2024) and this study	​
Custom-written MATLAB code for quantifying neuronal morphology	Donovan et al. (2024) and this study	​

### *Drosophila* whole-brain dissection and immunostaining

Whole brains were dissected from pupae and adult female flies in cold 2% PFA supplemented with 0.1 M L-lysine, followed by fixation for 1 h on ice; pupal brains were briefly (∼1 min) rinsed in PBS with 1% Triton prior to fixation. After fixation, all brains (adult and pupal) were washed using PBST (PBS with 0.5% Triton) three times at 1-min intervals and blocked in PBST-NGS (PBST with 5% normal goat serum, Abcam) for 30 min at room temperature. Brains were then incubated in primary antibodies diluted in PBST-NGS (chicken anti-GFP, 1:1,000 dilution; rabbit anti-RFP, 1:50; and mouse anti-BRP, 1:10) for 48 h at 4°C. Brains were then washed three times at 30-min intervals with PBST-NGS before incubation with secondary antibodies (Alexa 488 goat anti-chicken, 1:1,000; Alexa 555 goat anti-rabbit, 1:500; and Alexa 647 goat anti-mouse, 1:500) for 24 h at 4°C. After staining, brains were washed with PBST-NGS three times at 20-min intervals and mounted on microscope slides. Each brain was positioned in 15 μl of SlowFade Gold Antifade (Invitrogen) in an iSpacer (Sun Jin Lab) with the posterior side of the optic lobes oriented toward the coverslip.

Immunostained brains were imaged using either a Leica SP8 confocal microscope (for reconstructions in Fig. 3, F–I) equipped with a 63× 1.4 NA oil immersion objective (Leica) or a confocal Zeiss LSM 900 (all other images) equipped with an Airyscan detector (Zeiss) and a 63× 1.4 NA oil immersion objective (Zeiss). For samples imaged with the Leica, overview z-stacks of the entire lobula plate (voxel size: 137 nm × 137 nm × 500 nm) were acquired for each sample, along with higher resolution z-stacks of somas and primary and distal dendrites (voxel size: 49.1 nm × 49.1 nm × 500 nm). On the Zeiss, overview z-stacks (voxel size: 49.5 nm × 49.5 nm × 500 nm) and higher resolution z-stacks of the subcellular compartments (voxel size: 42.7 nm × 42.7 nm × 150 nm) were collected using Airyscan Multiplex mode with 2× line averaging.

### *In vivo* imaging

Female flies were anesthetized on ice and positioned in a keyhole cut in a thin metal shim. Flies were positioned such that the back of the head was flush with the shim and the eyes were below the shim. Flies were then secured with UV-cured glue (Bondic) and dissected in a cold sugar saline solution (103 mM NaCl, 3 mM KCl, 5 mM TES, 1 mM NaH2PO4, 26 mM NaHCO2, 4 mM MgCl2, 1.5 mM CaCl2, 10 mM trehalose, 10 mM glucose, and 7 mM sucrose, bubbled with carbogen to reach a pH of 7.2). Fine forceps were used to create a small opening in the cuticle and remove fat and trachea above the left optic lobe. Brains were then perfused with room-temperature saline and imaged using an integrated confocal and two-photon microscope (Leica SP8 CSU MP Dual) equipped with a 20× 1.0 NA water immersion objective (Leica) and a resonant scan head.

For confocal imaging of mitochondrial transport, mitoGFP and a cytoplasmic red fluorescent protein (tdTomato) were selectively expressed in HS neurons using the R27B03-GAL4 driver line. To facilitate resolution of individual motile mitochondria, stationary mitochondria within the primary HS dendrite were photobleached using a 488-nm laser at 100% power for ∼30 s. Confocal z-stacks (voxel size = 108.5 nm × 108.5 nm × 1 μm; field of view = 26.1 μm × 26.1 μm × ∼13 μm) were collected at five-second intervals for 5 to 10 min.

For confocal imaging of mitochondrial mixing, mtDendra2 and a far-red fluorescent protein expressed in the cytosol (mIFP) were selectively expressed in HS neurons. Dendra2-labeled mitochondria within a 10.4 μm × 10.4 μm ROI were converted from green to red fluorescence by illumination with a 405-nm laser at 15–20% power for ∼30 s. Confocal z-stacks (voxel size = 108.5 nm × 108.5 nm × 1 μm; field of view = 56.2 μm × 56.2 μm × ∼30 μm) centered on the ROI were imaged immediately before and after photoconversion, as well as three h after photoconversion. Samples were kept in the dark in a 25C incubator between time points. To observe individual fusion events (Fig. S2), time-lapse images of photoconverted ROIs were collected at 10-min intervals for 90 min.

For two-photon imaging of visual stimulus-driven signals, samples were simultaneously excited with a tunable MP laser set to 920 nm and a fixed 1,045 laser line (Spectra-Physics Insight X3 Dual) to image ATP and calcium responses (iATPSnFR2 and jRGECO1a) or changes in pH (pHerry: SE-pHluorin and mCherry), or samples were excited at 920 nm alone to image either calcium (GCaMP6f) or voltage (JEDI-2P) responses. Images (pixel size: ∼130 nm × 130 nm; field of view: ∼26 μm × ∼13 μm) were collected at 100-ms intervals for 1–3 min. Multiple (∼2–5) distal dendrites were imaged per fly; total imaging time per fly was ∼30 min.

### Visual stimulus presentation

Visual stimuli were created using PsychoPy and displayed via a digital light projector (DLP LightCrafter, Texas Instruments). The stimuli were projected onto a screen positioned ∼2 cm away from the fly’s eye, covering ∼60° of its visual field horizontally and ∼60° vertically. To prevent the microscope from detecting light emitted by the projector, a 472/30-nm band-pass filter (Semrock) was used to filter the stimulus before it was projected onto the screen. The stimulus was refreshed at 60 Hz. To synchronize the stimuli and the imaging frames, voltage signals from the microscope were relayed to PsychoPy through a LabJack device. Full-contrast wide-field square-wave gratings (λ = 30°) that spanned the entire stimulus screen and moved in either the PD or ND at 30°/s were projected on the stimulus screen. For iATPSnFR2, jRGECO1a, and pHerry experiments, moving gratings were presented for 2 s, with static gratings presented for 2 s before and after motion; for JEDI-2P experiments, moving gratings were presented for 1 s, with static gratings presented for 0.5 s before and after. To measure the direction selectivity of calcium (GCaMP6f) responses, square-wave gratings moving in one of eight different directions, presented in a randomized order, were presented for 2 s, with static gratings presented for 2 s before and after motion.

### Image analysis

#### Rapid classification of mitochondrial morphology and dendrite degeneration

To quantify mitochondrial morphology, images of mitochondria (labeled with mitoGFP) in HS distal dendrites were scored by eye on a 5-point scale ranging from highly fragmented (1) to highly fused (5). To quantify dendrite degeneration, the fraction of neurons exhibiting severe dendrite fragmentation was scored based on images of HS distal dendrites (labeled with a cytosolic volume marker, tdTomato) for three independent replicates consisting of at least five optic lobes. In both cases, custom-written Python code was used to facilitate the scoring of randomly presented images in a gamified fashion. Players first worked through a tutorial that defined image classes (e.g., fragmented versus fused mitochondria and fragmented versus unfragmented dendrites) and indicated which keys to press for each image class. Then, players had to pass a graded trial run in which their scores for a subset of images were compared with gold standard scores (generated by the researcher who generated the images) before scoring all images. Images were stripped of identifying information (i.e., genotype) and presented in a randomized order. Each image was classified by at least four players, and final scores for each image were calculated by averaging the scores of all players.

#### Quantification of mitochondrial density

Mitochondrial density measurements were taken from 1 to 3 z-stacks per compartment (distal dendrites or somas) for each optic lobe. Automatic semantic segmentation of mitochondria, neuron, and background pixels was carried out using ilastik (Berg et al., 2019) and nnU-NET (Isensee et al., 2021). To generate training data, images were segmented in ilastik using pixel classification mode, allowing for labels of neuron, mitochondria, and background to be assigned to individual pixels. The segmented masks were then manually cleaned in ImageJ and used, along with the original images, to train an nnU-NET model. The trained nnU-NET model was then used to generate predictions for a larger set of images. When necessary, more masks were cleaned and fed into nnU-NET for additional rounds of training. Following successful prediction, segmentations were manually cleaned to remove background signals. Finally, mitochondrial density measurements—the total number of mitochondrial pixels divided by the sum of the mitochondrial plus neuron pixels—were extracted from segmented images using custom-written Python code.

#### Quantification of dendrite morphology

The number of HS dendrites was counted by manual scoring of the number of primary dendrites per optic lobe. To measure dendrite morphology, three-dimensional reconstructions of dendrites were generated based on a cytosolic volume marker (tdTomato) in a semi-manual fashion using neuTube (Feng et al., 2015). Before analysis, primary dendrite lengths were normalized to the shortest primary dendrite across all genotypes and ages using TREES Toolbox (Cuntz et al., 2011) in MATLAB. Total dendrite volume, total dendrite length, number of branch points, and parent–daughter and sister–sister scaling parameters were analyzed using previously published MATLAB and Python code (Donovan et al., 2024). To determine whether dendrite morphologies followed normal or bimodal distributions (Fig. S4), measurements of dendrite volume, length, and branch numbers were fit to Gaussian mixture models using the Mclust function in R.

#### Quantification of mitochondrial transport

Motile mitochondrial lengths, speeds, and linear flux rates were measured from maximum projections of confocal z-stack images of mitoGFP and cytosolic tdTomato, expressed in HS neurons. Prior to taking measurements, max projections of z-stack images were aligned using either the TurboReg Fiji plugin or custom-built Python code. Mitochondrial lengths were measured using the line tool in Fiji. Individual mitochondria were hand-tracked for both the anterograde and retrograde direction using the Tracking Fiji built in plugin. Measurements of pause-free mitochondrial speeds and linear flux rates were extracted from mitochondrial trajectories using custom-written Python code. Mitochondrial speeds were defined as the average speed (distance over time) above an instantaneous speed threshold of 0.1 μm/s. Linear flux rates were defined as the number of motile mitochondria moving through the primary dendrite in either the anterograde or retrograde direction per unit time. Volume fluxes ($F_{v})$, or total mitochondrial volumes moving through the primary dendrite per unit time, were calculated based on measurements of linear flux rates ($F_{l}$) and lengths of motile mitochondria (l), as well as a fixed mitochondrial radius (r = 0.3 μm, estimated from previous measurements of mitochondria in electron microscopy images of HS primary dendrites [Donovan et al., 2024]): $F_{v}=F_{l}\times \pi r^{2}l$. The time scale τ for mitochondrial replacement (i.e., the time required to replace the entire volume of mitochondria in an HS dendritic arbor via anterograde and retrograde transport alone) was estimated as a function of fly age based on previous measurements of the total mitochondrial volume in an HS dendrite ($V_{tot}$ = ∼400 μm^3^) and average volume flux measurements: $\tau =V_{tot}\;/F_{v}$.

#### Quantification of mitochondrial content exchange

*In vivo* images of mtDendra2 were analyzed to measure mitochondrial content exchange in primary and distal HS dendrites. Confocal z-stacks collected at two points after local photoconversion of mtDendra2-green to mtDendra2-red (immediately after photoconversion [t_0_] and 3 h later [t_3_]) were mutually aligned using custom-written Python code. Next, mitochondria were manually segmented within small ROIs within individual z-slices (2–3 slices per stack) using the paint tool in Fiji; segmentation was guided by careful visual inspection of the entire z-stack to avoid lumping pixels from multiple mitochondria into single ROIs. Then, using custom-written Python code, average green and red fluorescence intensities were extracted from each ROI, and the ratio of green (G) to red (R) fluorescence was calculated as (G-R)/(G+R). The change in this green–red ratio over time was calculated for photoconverted ROIs in each primary dendrite by subtracting the average green–red ratio for all mitochondria in the ROI at t_0_ from the average green–red ratio at t_3_. At each time point, variance in the green–red ratio was calculated according to $\sum {\left|GR_{i}-\bar{GR}\right|}^{2}/N$, where $GR_{i}$ is the green–red ratio for a manually segmented mitochondrion, $\bar{GR}$ is the average green–red ratio, and N is the number of segmented mitochondria in the photoconverted ROI.

#### Quantification of visual stimulus-driven responses

To measure stimulus-locked fluctuations in calcium (jRGECO1a or GCaMP6f), ATP (iATPSnFR2), voltage (JEDI-2P), or pH (pHerry) in HS dendrites, time series images were aligned using Python code based on the ImageJ TurboReg plugin. Then, ROIs were masked from the average projection of the time series via simple thresholding and manual clean-up in Fiji. Custom-written Python code was then used to measure pixel intensities within each ROI. To measure average responses to moving square-wave gratings, images were binned first by direction of motion (PD versus ND) and then by time (relative to the onset of motion); jRGECO1a, iATPSnFR2, and pHerry signals were sampled at 5 Hz, and GCaMP6f and JEDI-2P responses were sampled at 10 Hz. ΔF/F was calculated as (F(t)-F_0_)/F_0_, where F_0_ was the average fluorescence intensity in the 0.5 s immediately prior to the onset of motion. For pH measurements, pHerry ratios (R) were calculated by dividing the average SE-pHluorin (pH-sensitive) fluorescence intensity by the average mCherry (pH-insensitive) fluorescence intensity within each ROI at each time point, and ΔR/R was calculated as (R(t)-R_0_)/R_0_. For jRGECO1a and iATPSnFR2, response amplitudes were calculated by summing ΔF/F values in a three-second time window immediately following the onset of motion (this time window includes the two-second stimulus period, as well as the second immediately following the cessation of motion); for JEDI-2P, response amplitudes were calculated by summing ΔF/F values during the one-second stimulus period. The DSI was calculated for GCaMP6f responses according to $R_{0{}^{\circ}}/\sum_{\theta \in D}R_{\theta }$, where $R_{0{}^{\circ}}$ is the peak response (i.e., the maximum ΔF/F) to PD motion, and $\sum_{\theta \in D}R_{\theta }$ is the sum of the peak responses to all directions.

### RNAi validation

To validate the adult-restricted, RNAi-mediated KD of Milton or Marf, shRNAs were pan-neuronally expressed using the nSyb-GAL4 driver (GMR57C10-GAL4). Expression was suppressed during development using Gal80^ts^. Crosses were reared at 18°C, and adult flies were shifted to 29°C within 24 h of eclosion. Transcript levels were measured using qRT-PCR 7 days after the induction of the KD. For each biological replicate, 25 fly heads were separated from the body and homogenized in 500 μl of TRIzol reagent (Biomatik) for RNA extraction, followed by the addition of 100 μl chloroform and subsequent extraction steps per the manufacturer’s protocol. Samples were treated with DNase for RNA purification, and cDNA was synthesized using RevertAid First Strand cDNA Synthesis Kit (Thermo Fisher Scientific). Quantitative RT-PCR was performed on the StepOnePlus Applied Biosystems Real-Time PCR System using Applied Biosystems SYBR Green PCR Master Mix (Thermo Fisher Scientific). The transcript levels were normalized to the reference gene (Actin5C) and quantified relative to the control using the ΔΔCt method, providing relative fold change.

The following primer pairs (IDT Technologies) were used:

Actin5c-fwd- 5′-TTG​TCT​GGG​CAA​GAG​GAT​CAG-3′.

Actin5c-rev- 5′-ACC​ACT​CGC​ACT​TGC​ACT​TTC-3′.

Milton-fwd- 5′-GCA​GAC​GAT​GGC​ACA​GAT​ACT-3′.

Milton-rev- 5′-CGT​CGA​GCA​GGG​AGT​TGA​C-3′.

Marf-fwd-5′-ACTCATCGCTGCAACAATCC-3′.

Marf-rev-5′-ATCTGGAGCGGTGATTTGTCG-3′.

### Lifespan analysis

To analyze the effects of adult-restricted pan-neuronal or HS-specific Milton or Marf KD on fly longevity, crosses were reared at 18°C (to allow Gal80^ts^ to suppress RNAi expression) and adult flies were collected within 24 h of eclosion and reared in vials containing standard cornmeal molasses food. Every 2 days, flies were flipped into new vials and the number of surviving flies in each vial was recorded. For pan-neuronal KD experiments (Fig. S5 B), 16 vials containing 8 flies each were analyzed per genotype (for a total of 128 flies per genotype); for HS-specific KD experiments (Fig. S5 D), 10 vials containing 20 flies each were analyzed per genotype (a total of 200 flies per genotype).

### Climbing assay

Approximately 8 flies were put in each empty plastic vial and allowed to briefly acclimate. Flies were tapped to the bottom of the vial 3 times to initiate climbing, and videos were taken at 30fps with an iPhone13. A machine learning tool for animal tracking, SLEAP (Pereira et al., 2022), was used to track fly positions within the vial. Climbing data were recorded for 10 s. SLEAP was used to determine each fly’s normalized vertical position relative to the bottom of the vial at the 10-s time point. Flies were analyzed at two time points: 6–7 days and 13–15 days.

### Statistical analysis

Datasets were tested for normality using the Shapiro–Wilk test. For normally distributed data, single comparisons were performed using paired or unpaired *t* tests; multiple comparisons were performed using ANOVA and post hoc Tukey’s tests. For non-normal data, single comparisons were performed using Mann–Whitney U tests; multiple comparisons were performed using the Kruskal–Wallis test, followed by Dunn’s test with Benjamini–Hochberg P value adjustment. Lifespans (Fig. S5, B and D) were compared using the log-rank test.

The specific tests used for each dataset are indicated in the figure legends. In the figures, asterisks indicate significant differences for single comparisons, and letters indicate significant differences for multiple comparisons. Specifically, groups labeled with different letters are significantly different; groups labeled with the same letter are not significantly different. In Fig. 2 E, for example, anterograde flux rates at days 2 and 7 are significantly different and are thus labeled with two different letters (“A” and “B,” respectively). In contrast, there is no significant difference between retrograde flux at day 2 versus day 7, nor is there a significant difference in retrograde versus anterograde flux at either time point; thus, retrograde flux measurements at days 2 and 7 are labeled with letters common to all four groups (“AB”).

Analysis of bimodality of dendrite morphology measurements (Fig. S4 B) was conducted in R. All other statistical tests were implemented using the stats module in SciPy.

### Online supplemental material

Supplemental material includes five figures. Fig. S1 shows the R27B03-GAL4 driver line labels HS neurons in late pupation and adulthood. Fig. S2 shows mitochondrial fusion and content exchange in HS distal dendrites. Fig. S3 shows Milton KD has no effect on HS dendrite scaling. Fig. S4 shows Marf KD has a variable effect on HS dendrite size. Fig. S5 shows effects of adult-restricted perturbations of mitochondrial dynamics on fly longevity, locomotion, and HS dendrite function in young flies.

## Data Availability

All experimental measurements presented in this study are publicly available at https://github.com/BarnhartLabCU/Wang-et-al-2026-Data. Raw images are available from the corresponding author upon reasonable request.
